# Evaluation of Machine-Learning Algorithms for Predicting Opioid Overdose Risk Among Medicare Beneficiaries With Opioid Prescriptions

**DOI:** 10.1001/jamanetworkopen.2019.0968

**Published:** 2019-03-22

**Authors:** Wei-Hsuan Lo-Ciganic, James L. Huang, Hao H. Zhang, Jeremy C. Weiss, Yonghui Wu, C. Kent Kwoh, Julie M. Donohue, Gerald Cochran, Adam J. Gordon, Daniel C. Malone, Courtney C. Kuza, Walid F. Gellad

**Affiliations:** 1 Department of Pharmaceutical Outcomes & Policy, College of Pharmacy, University of Florida, Gainesville; 2 Department of Mathematics, University of Arizona, Tucson; 3 Carnegie Mellon University, Heinz College, Pittsburgh, Pennsylvania; 4 Department of Health Outcomes & Biomedical Informatics, University of Florida, College of Medicine, Gainesville; 5 Division of Rheumatology, Department of Medicine, and the University of Arizona Arthritis Center, University of Arizona, Tucson; 6 Department of Health Policy and Management, Graduate School of Public Health, University of Pittsburgh, Pittsburgh, Pennsylvania; 7 Program for Addiction Research, Clinical Care, Knowledge, and Advocacy, Division of Epidemiology, Department of Internal Medicine, University of Utah, Salt Lake City; 8 Informatics, Decision-Enhancement, and Analytic Sciences Center, Veterans Affairs Salt Lake City Health Care System, Salt Lake City, Utah; 9 Department of Pharmacy, Practice and Science, College of Pharmacy, University of Arizona, Tucson; 10 Center for Pharmaceutical Policy and Prescribing, Health Policy Institute, University of Pittsburgh, Pittsburgh, Pennsylvania; 11 Department of Medicine, School of Medicine, University of Pittsburgh, Pittsburgh, Pennsylvania; 12 Center for Health Equity Research Promotion, Veterans Affairs Pittsburgh Healthcare System, Pittsburgh, Pennsylvania

## Abstract

**Importance:**

Current approaches to identifying individuals at high risk for opioid overdose target many patients who are not truly at high risk.

**Objective:**

To develop and validate a machine-learning algorithm to predict opioid overdose risk among Medicare beneficiaries with at least 1 opioid prescription.

**Design, Setting, and Participants:**

A prognostic study was conducted between September 1, 2017, and December 31, 2018. Participants (n = 560 057) included fee-for-service Medicare beneficiaries without cancer who filled 1 or more opioid prescriptions from January 1, 2011, to December 31, 2015. Beneficiaries were randomly and equally divided into training, testing, and validation samples.

**Exposures:**

Potential predictors (n = 268), including sociodemographics, health status, patterns of opioid use, and practitioner-level and regional-level factors, were measured in 3-month windows, starting 3 months before initiating opioids until loss of follow-up or the end of observation.

**Main Outcomes and Measures:**

Opioid overdose episodes from inpatient and emergency department claims were identified. Multivariate logistic regression (MLR), least absolute shrinkage and selection operator–type regression (LASSO), random forest (RF), gradient boosting machine (GBM), and deep neural network (DNN) were applied to predict overdose risk in the subsequent 3 months after initiation of treatment with prescription opioids. Prediction performance was assessed using the C statistic and other metrics (eg, sensitivity, specificity, and number needed to evaluate [NNE] to identify one overdose). The Youden index was used to identify the optimized threshold of predicted score that balanced sensitivity and specificity.

**Results:**

Beneficiaries in the training (n = 186 686), testing (n = 186 685), and validation (n = 186 686) samples had similar characteristics (mean [SD] age of 68.0 [14.5] years, and approximately 63% were female, 82% were white, 35% had disabilities, 41% were dual eligible, and 0.60% had at least 1 overdose episode). In the validation sample, the DNN (C statistic = 0.91; 95% CI, 0.88-0.93) and GBM (C statistic = 0.90; 95% CI, 0.87-0.94) algorithms outperformed the LASSO (C statistic = 0.84; 95% CI, 0.80-0.89), RF (C statistic = 0.80; 95% CI, 0.75-0.84), and MLR (C statistic = 0.75; 95% CI, 0.69-0.80) methods for predicting opioid overdose. At the optimized sensitivity and specificity, DNN had a sensitivity of 92.3%, specificity of 75.7%, NNE of 542, positive predictive value of 0.18%, and negative predictive value of 99.9%. The DNN classified patients into low-risk (76.2% [142 180] of the cohort), medium-risk (18.6% [34 579] of the cohort), and high-risk (5.2% [9747] of the cohort) subgroups, with only 1 in 10 000 in the low-risk subgroup having an overdose episode. More than 90% of overdose episodes occurred in the high-risk and medium-risk subgroups, although positive predictive values were low, given the rare overdose outcome.

**Conclusions and Relevance:**

Machine-learning algorithms appear to perform well for risk prediction and stratification of opioid overdose, especially in identifying low-risk subgroups that have minimal risk of overdose.

## Key Points

**Question:**

Can machine-learning approaches predict opioid overdose risk among fee-for-service Medicare beneficiaries?

**Findings:**

In this prognostic study of the administrative claims data of 560 057 Medicare beneficiaries, the deep neural network and gradient boosting machine models outperformed other methods for identifying risk, although positive predictive values were low given the low prevalence of overdose episodes.

**Meaning:**

Machine-learning algorithms using administrative data appear to be a valuable and feasible tool for more accurate identification of opioid overdose risk.

## Teaser

This prognostic study evaluates the use of machine-learning methods, with prescription drug and claims data, in detecting opioid overdose risk in Medicare beneficiaries with at least 1 opioid prescription.

## Introduction

In 2016, 11.8 million American individuals reported using prescription opioids nonmedically,^1^ and an estimated 115 individuals died each day from opioid overdose.^2,3,4^ The annual cost of misuse or abuse of opioids exceeds $78.5 billion, including the costs of health care, lost productivity, substance abuse treatment, and the criminal justice system.^5^

In response, health systems, payers, and policymakers have developed programs to identify and intervene in individuals at high risk of problematic opioid use and overdose. These programs, whether outreach calls from case managers, prior authorizations, referrals to substance use disorder specialists, dispensing of naloxone hydrochloride, or enrollment in lock-in programs, can be expensive to payers and burdensome to patients. The determination of who is at high risk is a factor in the size and scope of these interventions and the resources expended. Yet, the definition of high risk is variable, ranging from a high-dose opioid (defined using various cut points) to the number of pharmacies or prescribers that a patient visits. These criteria, for example, determine how Medicare beneficiaries are selected into so-called lock-in programs in Medicare, also called the Comprehensive Addiction and Recovery Act (CARA) drug management programs.^6^ These programs will soon be required for all Part D plans.^7^

These current measures of high risk were derived from studies that used traditional statistical methods to identify risk factors for overdose rather than predict an individual’s risk.^8,9,10,11,12,13,14,15,16,17,18,19,20,21,22,23,24,25,26,27,28,29,30,31^ However, individual risk factors may not be strong predictors of overdose risk.^32^ Moreover, traditional statistical approaches have limited ability to handle nonlinear risk prediction and complex interactions among predictors. For example, receipt of a high-dose opioid is a well-known overdose risk factor, but the complex interactions between opioid dose, substance use disorders, mental health, emergency department visits, prescriber characteristics, and socioeconomic variables may yield greater predictive power than one factor alone. The few previous studies focused on predicting opioid overdose (rather than simply identifying risk factors) either had suboptimal prediction performance^24,28,31^ or used case-control designs that were unable to measure true overdose incidence and may not adequately calibrate algorithms to real data for rare outcomes such as overdose.^22,25,30^

Machine learning is an alternative analytic approach to handling complex interactions in large data, discovering hidden patterns, and generating actionable predictions in clinical settings. In many cases, machine learning is superior to traditional statistical techniques.^33,34,35,36,37,38^ Machine learning has been widely used in activities from fraud detection to genomic studies but, to our knowledge, has not yet been applied to address the opioid epidemic. Our overall hypothesis was that a machine-learning algorithm would perform better in predicting opioid overdose risk compared with traditional statistical approaches.

The objective of this study was to develop and validate a machine-learning algorithm to predict opioid overdose among Medicare beneficiaries with at least 1 opioid prescription. Based on the prediction score, we stratified beneficiaries into subgroups at similar overdose risk to support clinical decisions and improved targeting of intervention. We chose Medicare because of the high prevalence of prescription opioid use and the availability of national claims data and because the program will require specific interventions targeting individuals at high risk for opioid-associated morbidity.^6,7^

## Methods

### Design and Sample

This prognostic study was conducted between September 1, 2017, and December 31, 2018. The University of Arizona Institutional Review Board approved the study. This study followed the Standards for Reporting of Diagnostic Accuracy (STARD) and the Transparent Reporting of a Multivariable Prediction Model for Individual Prognosis or Diagnosis (TRIPOD) reporting guidelines.^39,40^

We included prescription drug and medical claims for a 5% random sample of Medicare beneficiaries between January 1, 2011, and December 31, 2015. We identified fee-for-service adult beneficiaries without cancer who were US residents and received 1 or more opioid prescriptions during the study period. We excluded beneficiaries who (1) filled only parenteral opioid prescriptions and/or cough or cold medication prescriptions containing opioids, (2) had malignant cancer diagnoses (eTable 1 in the Supplement), (3) received hospice, (4) ever enrolled in Medicare Advantage plans (because their health care use in Medicare Advantage may not be observable), or (5) had their first opioid prescription after October 1, 2015 (eFigure 1 in the Supplement). An index date was defined as the date of a patient’s first opioid prescription between April 1, 2011, and September 30, 2015. Once eligible, beneficiaries remained in the cohort, regardless of whether they continued to receive opioid prescriptions, until they were censored because of death or the end of observation.

### Outcome Variables: Opioid Overdose

We identified any occurrence of fatal or nonfatal opioid overdose (prescription opioids or other opioids, including heroin), defined in each 3-month window after the index prescription using the *International Classification of Diseases, Ninth Revision*, and *International Statistical Classification of Diseases and Related Health Problems, Tenth Revision (ICD-10)*, codes for overdose (eTable 2 in the Supplement) from inpatient or emergency department settings.^14,41,42,43,44^ Overdose was defined with either an opioid overdose code as the primary diagnosis (80% of identified overdose episodes) or other drug overdose or substance use disorder code as the primary diagnosis (eTable 3 in the Supplement) and opioid overdose as the nonprimary diagnosis (20% of identified overdose episodes), as defined previously.^14^ Sensitivity analyses using opioid overdose as the primary diagnosis and capturing any opioid overdose diagnosis code in any position yielded similar results.

### Predictor Candidates

We compiled 268 predictor candidates, informed by the literature (eTable 4 in the Supplement).^8,9,10,11,12,13,14,15,16,17,18,19,20,21,22,23,24,25,26,27,28,29,30,31^ Patient, practitioner, and regional factors were measured at baseline in the 3 months before the first opioid prescription fill and in 3-month windows after initiating prescription opioids. We chose a 3-month window in accordance with the literature and to be consistent with the quarterly evaluation period commonly used by prescription drug monitoring programs and health plans.^13,14,45^ In the primary analysis, we used the variables measured in each 3-month period (eg, the first) to predict overdose risk in each subsequent 3-month period (eg, the second) (eFigure 2A in the Supplement). In sensitivity analyses, instead of using a previous 3-month period to predict overdose in the next period, we included information collected in all of the historical 3-month windows to predict opioid risk for each 3-month period for each person (eFigure 2B in the Supplement).

The predictor candidates also included a series of variables related to prescription opioid and relevant medication use: (1) total and mean daily morphine milligram equivalent (MME),^17^ (2) cumulative and continuous duration of opioid use (ie, no gap >32 days between fills),^45^ (3) total number of opioid prescriptions overall and by active ingredient, (4) type of opioid based on the US Drug Enforcement Administration’s Controlled Substance Schedule (I-IV) and duration of action, (5) number of opioid prescribers, (6) number of pharmacies providing opioid prescriptions,^11,17,23^ (7) number of early opioid prescription refills (refilling opioid prescriptions >3 days before the previous prescription runs out),^46^ (8) cumulative days of early opioid prescription refills, (9) cumulative days of concurrent benzodiazepines and/or muscle relaxant use, (10) number and duration of other relevant prescriptions (eg, gabapentinoids), and (11) receipt of methadone hydrochloride or buprenorphine hydrochloride for opioid use disorder.^19,47,48,49,50^

Patient sociodemographic characteristics included age, sex, race/ethnicity, disability as the reason for Medicare eligibility, receipt of low-income subsidy, and urbanicity of county of residence. Health status factors (eg, number of emergency department visits) were derived from the literature and are listed in eTable 4 in the Supplement.^13,16,51,52,53,54^ Practitioner factors included opioid prescriber’s sex, specialty, mean monthly opioid prescribing volume and MME, and mean monthly number of patients receiving opioids. Many beneficiaries had more than 1 opioid prescriber, in which case the practitioner prescribing the highest number of opioids was designated as the primary prescriber. Regional factors (eg, percentage of households below the federal poverty level) included variables obtained from publicly available resources, including the Area Health Resources Files, Area Deprivation Index data sets,^55^ and County Health Rankings data.^56^

### Machine-Learning Approaches and Prediction Performance Evaluation

Our primary goal was risk prediction, and the secondary goal was risk stratification (ie, identifying patient subgroups at similar overdose risk). First, we randomly and equally divided the cohort into training (developing algorithms), testing (refining algorithms), and validation (evaluating algorithm’s prediction performance) samples. In both the primary and sensitivity analyses (eFigure 2 in the Supplement), we developed and tested prediction algorithms for opioid overdose using 5 commonly used machine-learning approaches: multivariate logistic regression, least absolute shrinkage and selection operator–type regression (LASSO), random forest (RF), gradient boosting machine (GBM), and deep neural network (DNN). Previous studies consistently showed that these methods yield the best prediction results^57,58^; the eAppendix in the Supplement describes the details for each approach used. Given that beneficiaries may have multiple opioid overdose episodes, we present the results from a patient-level random subset (ie, using one 3-month period with predictor candidates measured to predict risk in the subsequent 3 months for each patient) from the validation data for ease of interpretation. Episode-level performance was the same as the patient-level results.

To assess discrimination performance (ie, the extent to which patients who were predicted to be high risk exhibited higher overdose rates compared with those who were predicted to be low risk), we compared the C statistic (or area under the receiver operating curve) and precision-recall curves^59^ across different methods from the validation sample using the DeLong Test.^60^ Given that overdose events are rare outcomes and C statistics do not incorporate information about outcome prevalence, we reported other metrics, including sensitivity, specificity, positive predictive value (PPV), negative predictive value (NPV), positive likelihood ratio, negative likelihood ratio, number needed to evaluate (NNE) to identify 1 overdose episode, and estimated rate of alerts, to thoroughly assess the algorithms’ prediction ability (eFigure 3 in the Supplement).^61,62^ To compare performance across methods, we presented and assessed these metrics at the optimized prediction threshold that balances sensitivity and specificity, as identified by the Youden index.^63^ Furthermore, because no single threshold is suitable for every purpose, we also presented these metrics at multiple other levels of sensitivity and specificity (eg, arbitrarily choosing 90% sensitivity) to enable risk-benefit evaluations of potential interventions that use different thresholds defining high risk.

On the basis of the distribution of individuals’ estimated probability of an overdose event, we classified beneficiaries in the validation sample into low risk (predicted score below the optimized threshold), medium risk (score between the optimized threshold and top fifth percentile), or high risk (the top fifth percentile of scores, chosen according to clinical utility). We evaluated calibration plots (the extent to which the predicted overdose risk agreed with the observed risk) by the 3 risk groups.

To ensure clinical utility, we reported the predictors with the strongest effect. Because no standardized methods exist to identify individual important predictors from the DNN model, we reported the top 50 important predictors from the GBM and RF models. We also compared our prediction performance over a 12-month period with any of the 2019 Centers for Medicare & Medicaid Services opioid safety measures, which are meant to identify high-risk individuals or utilization behavior in Medicare.^64^ These simpler decision metrics were constructed from factors identified from previous studies using traditional approaches (eg, multivariate logistic regression). These measures included 3 metrics: (1) high-dose use, defined as higher than 120 MME for 90 or more continuous days; (2) 4 or more opioid prescribers and 4 or more pharmacies; and (3) concurrent opioid and benzodiazepine use for 30 or more cumulative days. In addition to using 3- and 12-month windows, we conducted a sensitivity analysis using a 6-month window in DNN to examine whether the prediction quality changes with different time horizons.^65^

### Statistical Analysis

We compared the patient characteristics by overdose status and by training, testing, and validation sample with unpaired, 2-tailed *t* test, χ^2^ test and analysis of variance, or corresponding nonparametric tests, as appropriate. We assessed correlations between 2 variables using Pearson correlation coefficient (*r*). Statistical significance was defined as 2-tailed *P* < .05.

All analyses were performed using SAS, version 9.4 (SAS Institute Inc); Python, version 3.6 (Python Software Foundation); and Salford Predictive Modeler software suite, version 8.2 (Salford System).

## Results

### Patient Characteristics

Beneficiaries in the training (n = 186 686), testing (n = 186 685), and validation (n = 186 686) samples had similar characteristics and outcome distributions (approximately 63% were female, 82% were white, 35% had disabilities, and 41% were dual eligible; the mean [SD] age was 68.0 [14.5] years (eTable 5 in the Supplement). Overall, 3188 beneficiaries (0.6%) had at least 1 opioid overdose episode during the study period.

### Prediction Performance of Machine-Learning Algorithms

**Figure 1.**
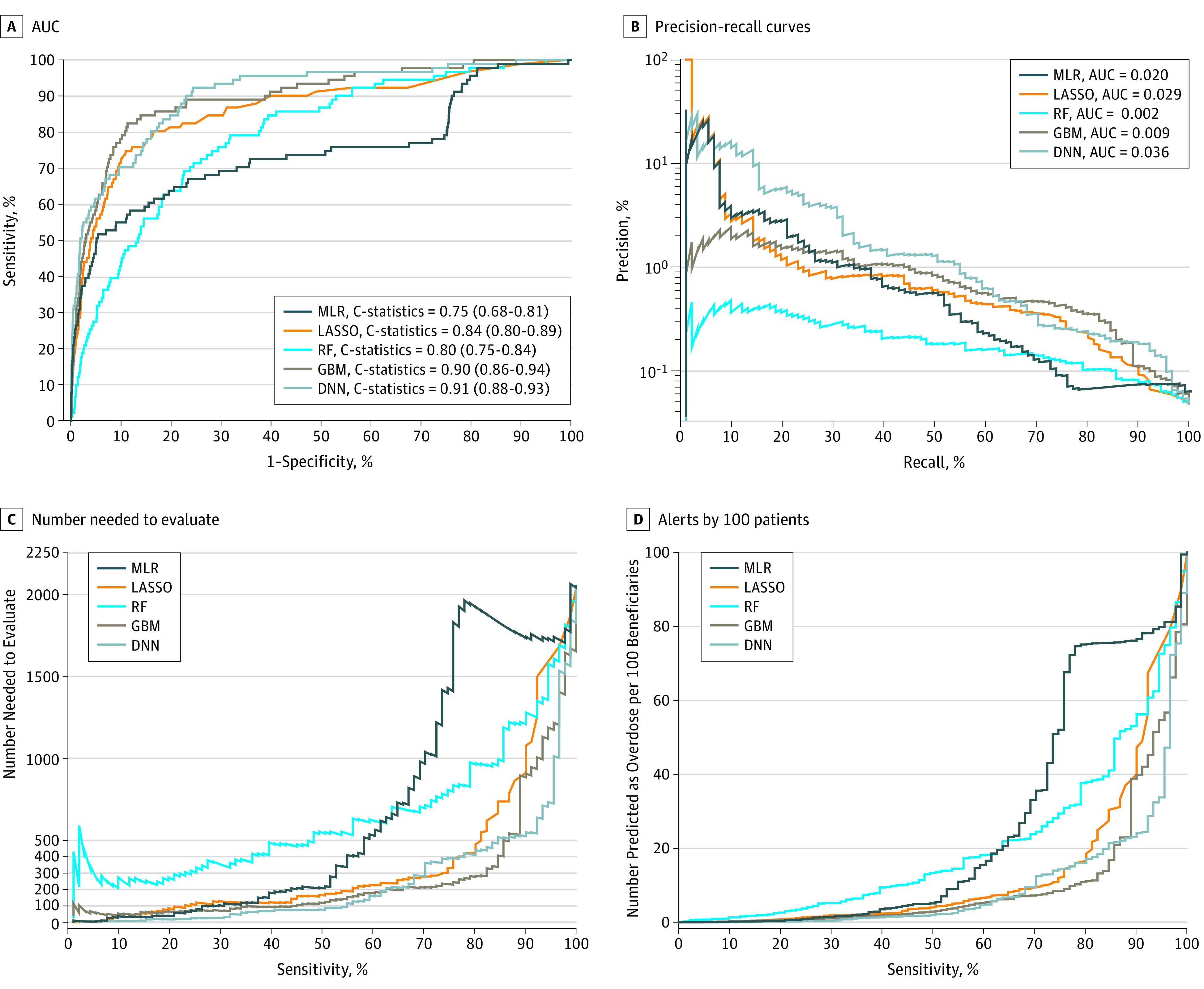
Performance Matrix of Machine-Learning Models for Predicting Opioid Overdose in Medicare Beneficiaries The 4 prediction performance matrixes in the validation sample are the area under the receiver operating characteristic curve (AUC) or C statistic (A); the precision-recall curves, which have improved performance if they are closer to the upper right corner or above the other method (B); the number needed to evaluate (NNE) by different cutoffs of sensitivity (C); and alerts per 100 patients by different cutoffs of sensitivity (D). DNN indicates deep neural network; GBM, gradient boosting machine; LASSO, least absolute shrinkage and selection operator–type regularized regression; MLR, multivariate logistic regression; and RF, random forest.

Figure 1 summarizes 4 prediction performance measures of each model. The DNN (C statistic = 0.91; 95% CI, 0.88-0.93) and GBM (C statistic = 0.90; 95% CI, 0.87-0.94) algorithms outperformed the LASSO (C statistic = 0.84; 95% CI, 0.80-0.89), RF (C statistic = 0.80; 95% CI, 0.75-0.84), and multivariate logistic regression (C statistic = 0.75; 95% CI, 0.69-0.80) methods for predicting opioid overdose (*P* < .001). In addition, DNN and GBM had similar prediction performance, and DNN had the best precision-recall performance (Figure 1B), based on an area under the curve of 0.036. Sensitivity analyses including all the historical 3-month windows yielded similar results (eFigure 4 in the Supplement).

eTable 6 in the Supplement shows the prediction performance measures across different levels (90%-100%) of sensitivity and specificity for each method. At the optimized sensitivity and specificity, as measured by the Youden index, GBM had a sensitivity of 86.8%, specificity of 81.1%, PPV of 0.22%, NPV of 99.9%, NNE of 447, and 24 positive alerts per 100 beneficiaries. Similarly, at the optimized sensitivity and specificity, DNN had a sensitivity of 92.3%, specificity of 75.7%, PPV of 0.18%, NPV of 99.9%, NNE of 542, and 22 positive alerts per 100 beneficiaries (Figure 1C and D; eTable 6 in the Supplement). If sensitivity were instead set at 90% (ie, attempting to identify 90% of individuals with actual overdose episodes), GBM had a specificity of 72.3%, PPV of 0.16%, NPV of 99.9%, NNE of 631 to identify 1 overdose, and 28 positive alerts generated per 100 beneficiaries; DNN had a specificity of 77.0%, PPV of 0.19%, NPV of 99.9%, NNE of 525, and 23 positive alerts per 100 beneficiaries (eTable 6 in the Supplement). If specificity were set at 90% (ie, identifying 90% of individuals with actual nonoverdose), GBM had a sensitivity of 74.7%, PPV of 0.41%, NPV of 99.9%, NNE of 245, and 9 positive alerts per 100 beneficiaries; DNN had a sensitivity of 70.3%, PPV of 0.34%, and NPV of 99.9%, NNE of 294, and 10 positive alerts per 100 beneficiaries. Overall, DNN’s prediction scores were highly correlated with the GBM’s prediction scores (*r = *0.73 for all patients, 0.73 for those without overdose episodes, 0.80 for those with overdose episodes; eFigure 5 in the Supplement).

### Risk Stratification Using Predicted Probability 

**Figure 2.**
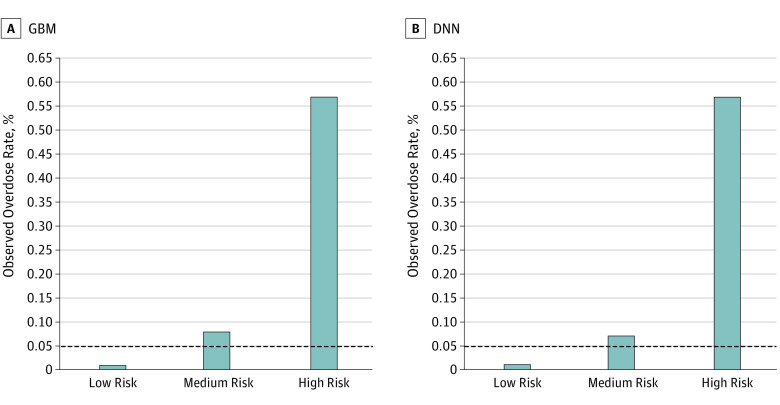
Calibration Performance of Gradient Boosting Machine (GBM) and Deep Neural Network (DNN) by Risk Group Risk subgroups were classified into 3 groups using the optimized threshold in the validation sample (n = 186 686): low risk (score below the optimized threshold), medium risk (predicted score between the optimized threshold, identified by the Youden index, and the top fifth percentile score), and high risk (predicted score in the top fifth percentile). The dashed line indicates the overall observed overdose rate without risk stratifications.

Using the GBM algorithm, 144 860 (77.6%) of the sample were categorized into low risk, 32 415 (17.4%) into medium risk, and 9411 (5.0%) into high risk for overdose (Table 1). Among all 91 beneficiaries with an overdose episode in the sample, 54 (59.3%) were captured in the high-risk group. Similarly, using the DNN algorithm, 9747 individuals (5.2%) were predicted to be high risk, capturing 56 overdose episodes (61.5%). Among the 142 180 individuals (76.2%) categorized as low risk, 99.99% did not have an overdose. Figure 2 depicts the actual overdose rate for individuals in each of the 3 risk groups. Across both the GBM and DNN models, those in the high-risk group had 7 to 8 times the risk of overdose compared with those in the lower-risk groups (observed overdose rate of GBM: 0.57% [high risk], 0.08% [medium risk], and 0.01% [low risk]; observed overdose rate of DNN: 0.57% [high risk], 0.07% [medium risk], and 0.01% [low risk]). Again, depicted is the negligible rate of overdose in the low-risk subgroups, representing more than three-quarters of the sample.

**Table 1.** Prediction Performance of Gradient Boosting Machine and Deep Neural Network Models in the Validation Sample Divided Into Risk Subgroups^a^

Performance Metric	GBM			DNN		
	Low Risk	Medium Risk	High Risk	Low Risk	Medium Risk	High Risk
Total, No. (%)	144 860 (77.6)	32 415 (17.4)	9411 (5.0)	142 180 (76.2)	34 759 (18.6)	9747 (5.2)
Predicted score, median (range)^b^	14.6 (1.4-39.0)	55.4 (39.0-77.7)	83.8 (77.7-93.8)	14.2 (2.1-46.5)	61.6 (46.5-81.9)	88.7 (81.9-99.7)
No. of actual overdose episodes (% of each subgroup)	11 (0.01)	26 (0.08)	54 (0.57)	9 (0.01)	26 (0.07)	56 (0.57)
No. of actual nonoverdose episodes (% of each subgroup)	144 849 (99.99)	32 389 (99.92)	9357 (99.43)	142 171 (99.99)	34 733 (99.93)	9691 (99.43)
Sensitivity, %	0	100	100	0	100	100
PPV, %^c^	NA	0.08	0.57	NA	0.07	0.57
NNE^c^	NA	1247	174	NA	1337	174
Specificity, %	100	0	0	100	0	0
NPV, %^c^	99.99	NA	NA	99.99	NA	NA
Overall No. of misclassified overdose episodes (% of overall cohort)^c^	11 (0.006)	32 389 (17.4)	9357 (5.0)	9 (0.005)	34 733 (18.6)	9691 (5.2)
% of All overdose episodes captured over 3 mo (n = 91)	12.1	29.6	59.3	9.9	28.6	61.5

Abbreviations: DNN, deep neural network; GBM, gradient boosting machine; NA, not able to be calculated owing to 0 denominator; NNE, number needed to evaluate; NPV, negative predictive value; PPV, positive predictive value.

^a^ Risk subgroups were classified into low risk (score below the optimized threshold), medium risk (predicted score between the optimized threshold and the top fifth percentile score), and high risk (predicted score in the top fifth percentile). The optimized thresholds were 39 (or probability of 0.39) for GBM and 46.5 (or probability of 0.465) for DNN.

^b^ Predicted scores were calculated by the predicted probability of overdose multiplied by 100.

^c^ If classifying medium- and high-risk groups as overdose and low-risk group as nonoverdose, then the PPV and NNE were not able to be calculated for the low-risk group because this group was considered as nonoverdose. Similarly, the NPV was not able to calculate for the medium- and high-risk groups because these groups were considered as overdose. Detailed definitions of prediction performance metrics are provided in eFigure 3 in the Supplement.

**Figure 3.**
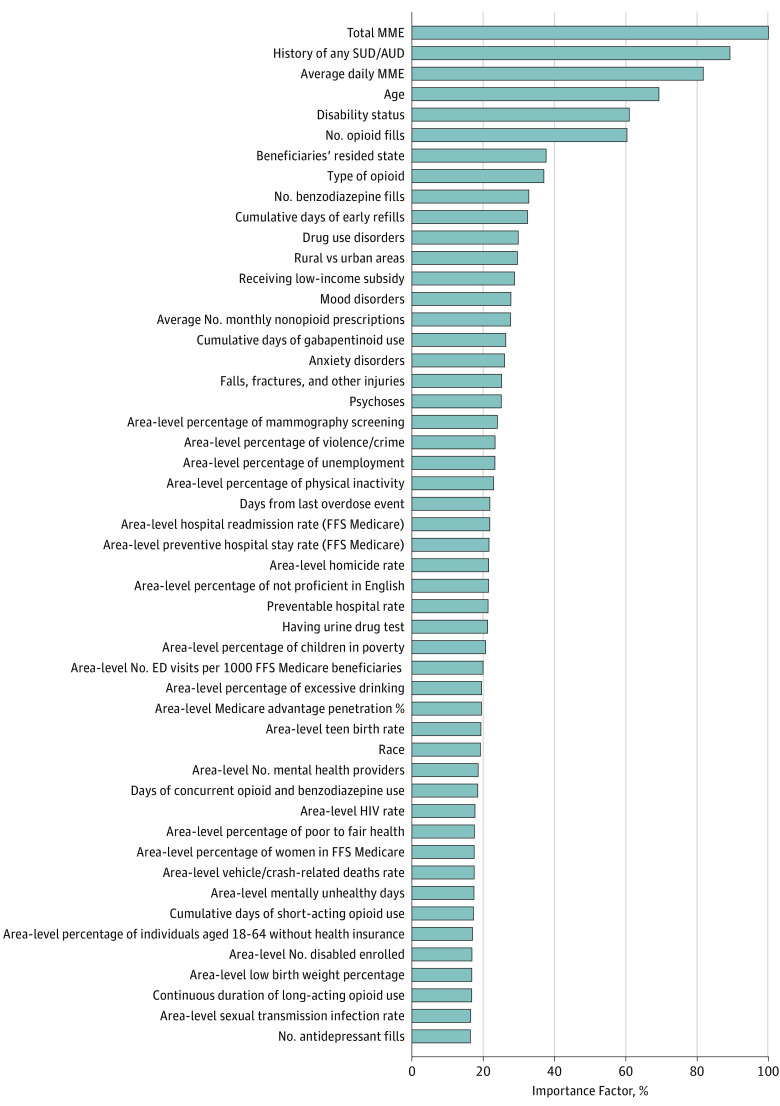
Top 50 Important Predictors for Opioid Overdose Selected by Gradient Boosting Machine Rather than *P* values or coefficients, the gradient boosting machine reports the importance of predictors included in a model. Importance is a measure of each variable’s cumulative contribution toward reducing square error, or heterogeneity within the subset, after the data set is sequentially split according to that variable. Thus, importance reflects a variable’s significance in prediction. Absolute importance is then scaled to give relative importance, with a maximum importance of 100. For example, the top 10 important predictors identified from the gradient boosting machine model included total opioid dose (eg, >1500 morphine milligram equivalent [MME] during 3 months), diagnosis of alcohol use disorders or substance use disorders (AUD/SUD), mean daily opioid dose (eg, >32 MME), age, disability status, total number of opioid prescriptions (eg, >4), beneficiary’s state residency (eg, Florida, Kentucky, or New Jersey), type of opioid use (eg, with mixed schedules), total number of benzodiazepine prescription fills (eg, >3), and cumulative days of early prescription refills (eg, >19 days). ED indicates emergency department; FFS: fee-for-service.

Figure 3 shows the most important predictors (n = 50) identified by the GBM model, such as total MME, history of any substance use disorder, mean daily MME, age, and Medicare disability status. eFigure 6 in the Supplement shows the most important predictors (n = 50) identified by the RF model.

Table 2 compares the performance of the DNN algorithm measures with the Centers for Medicare & Medicaid Services opioid safety measures. By targeting the high-risk group, DNN’s algorithm captured approximately 90% of 297 individuals with actual overdose episodes (NNE = 56) in a 12-month period, with 14 917 (8.9%) of the overall cohort being misclassified as having overdose, whereas the Centers for Medicare & Medicaid Services measures captured 30% of 297 individuals actual overdose episodes (NNE = 108), with 5.51% of the overall cohort being misclassified as overdose. The GBM and DNN algorithms performed similarly (eTable 7 in the Supplement). Sensitivity analyses using 6-month windows yielded similar C statistic with an improved PPV compared with using 3-month windows (eg, C statistic = 0.89; 95% CI, 0.87-0.90; PPV = 0.36% in DNN).

**Table 2.** Comparison of Prediction Performance Between Centers for Medicare & Medicaid Services Measures and Deep Neural Network Measures Over a 12-Month Period

Performance Metric	DNN Measures^a^			CMS Opioid Safety Measures^b^	
	Low Risk	Medium Risk	High Risk	Low- or No-Risk Opioid Use	High-Risk Opioid Use
Total, No. (%)	112 548 (67.5)	38 846 (23.3)	15 186 (9.1)	157 299 (94.4)	9281 (5.5)
Predicted score, median (range)	14.0 (2.1-46.5)	62.8 (46.5-81.9)	88.1 (81.9-99.7)	NA	NA
No. of actual overdose episodes (% of each subgroup)	7 (0.006)	21 (0.05)	269 (1.77)	210 (0.13)	87 (0.93)
No. of actual nonoverdose episodes (% of each subgroup)	112 541 (99.99)	38 825 (99.94)	14 917 (98.22)	157 089 (99.86)	9194 (99.06)
Sensitivity, %	0	100	100	0	100
PPV, %	NA	0.05	1.77	NA	0.93
NNE	NA	2000	56	NA	108
Specificity, %	100	0	0	100	0
NPV, %	99.99	NA	NA	99.86	NA
Overall No. of misclassified overdose episodes (% of overall cohort)^c^	7 (0.004)	38 825 (23.3)	14 917 (8.95)	210 (0.12)	9194 (5.51)
% of all overdose episodes captured over 12 mo (n = 297)	2.35	7.07	90.57	70.7	29.29

Abbreviations: CMS, Centers for Medicare & Medicaid Services; DNN, deep neural network; NA, not able to calculate; NNE, number needed to evaluate; NPV, negative predictive value; PPV, positive predictive value.

^a^ In contrast to Table 1, the measures were defined according to a 12-month period rather than a 3-month period. The sample size was smaller than in the main analysis because it required people to have at least 12 months of follow-up.

^b^ The 2019 CMS opioid safety measures are meant to identify high-risk individuals or utilization behavior.^64^ These measures include 3 metrics: (1) high-dose use, defined as higher than 120 morphine milligram equivalent (MME) for 90 or more continuous days, (2) 4 or more opioid prescribers and 4 or more pharmacies, and (3) concurrent opioid and benzodiazepine use for 30 or more days.

^c^ If classifying medium- and high-risk groups as overdose for DNN and low-risk group as nonoverdose, then individuals with actual nonoverdose in these 2 groups were misclassified. If classifying those with any of CMS high-risk opioid use measures as overdose, and the remaining group considered as nonoverdose, then individuals with actual nonoverdose in the high-risk groups were misclassified. The PPV and NNE were not able to calculate for the low-risk group because this group was considered as nonoverdose. Similarly, the NPV was not able to calculate for the medium- and high-risk groups because these groups were considered as overdose. Detailed definitions of prediction performance metrics are provided in eFigure 3 in the Supplement.

## Discussion

Using national Medicare data, we developed machine-learning models with strong performance for predicting opioid overdose. The GBM and DNN models achieved high C statistic (>0.90) for predicting overdose risk in the subsequent 3 months after initiation of treatment with prescription opioids and outperformed traditional classification techniques. As expected in a population with very low prevalence of the outcome, the PPV of the models was low; however, these algorithms effectively segmented the population into 3 risk groups according to predicted risk score, with three-quarters of the sample in a low-risk group with a negligible overdose rate and more than 90% of individuals with overdose captured in the high- and medium-risk groups. The ability to identify such risk groups has important potential for policymakers and payers who currently target interventions based on less accurate measures to identify patients at high risk.

We identified 7 previously published studies of opioid prediction models, each focused on predicting a different aspect of opioid use disorder and not applying advanced machine learning. The studies predicted a 12-month risk of opioid use disorder diagnosis using private insurance claims^13,22^; 2-year risk of clinical, electronic medical record–documented problematic opioid use in a primary care setting^24^; 12-month risk of overdose or suicide-associated events using data from the Veterans Health Administration ^31^; 6-month risk of serious prescription opioid–induced respiratory depression or overdose using data from the Veterans Health Administration and claims data from Insurance Management Services private insurance^25,30^; and 2-year risk of fatal or nonfatal overdose using electronic medical record data.^28^ These studies had several key limitations, including use of case-control designs unable to calibrate to population-level data with the true incidence rate of overdose; measuring predictors at baseline rather than over time; capturing only the first overdose episode; inability to identify complex or nonintuitive relationships (interactions) between the predictors and outcomes; and having suboptimal prediction performance (with a C statistic of up to 0.72 in non–case-control designs). The present study overcomes these limitations using a population-based sample and machine-learning methods. To our knowledge, this study is the first to predict overdose risk in the subsequent 3-month period after initiation of treatment with prescription opioids as opposed to 1-year or longer period.

The extant literature in predicting health outcomes often focuses on C statistics rather than the full spectrum of prediction performance. This study found high C statistics (>0.90) from machine-learning approaches. However, although opioid overdose represents a particularly important outcome, it is a rare outcome, especially in the Medicare population. Relying on C statistics alone may lead to overestimating the advantages of a prediction tool or underestimating the costs of clinical resources involved. For a preimplementation evaluation of a clinical prediction tool, it is recommended that researchers report sensitivity and at least 1 other metric (eg, PPV, NNE, or estimated alert rate) to present a more complete picture of the performance characteristics of a specific model.^59,61^ In this study, the NNE value using DNN and GBM algorithms is similar to other commonly used cancer screening tests, such as annual mammography to prevent 1 breast cancer death (NNE = 233-1316, varying by subgroups with different underlying risk).^66^

Unlike sensitivity and specificity, which are properties of the test alone, the PPV and NPV are affected by the prevalence of the outcome in the population tested. Low outcome prevalence leads to low PPV and high NPV, even in tests with high sensitivity and specificity, and could limit the clinical utility of a prediction algorithm such as ours because of false-positives. Other tests with good discrimination have low PPV because of overall prevalence, including trisomy 21 screening in 20- to 30-year-old women (prevalence of approximately 1:1200),^67^ with a PPV of 1.7% at a test with sensitivity higher than 99% and specificity higher than 95%. Despite the low PPV in this study, our risk stratification strategies may more efficiently guide the targeting of opioid interventions among Medicare beneficiaries compared with exisiting measures. This strategy first excludes most (approximately 75%) prescription opioid users with negligible overdose risk from burdensome interventions like pharmacy lock-in programs and specialty referrals. Targeting medium- and/or high-risk groups can capture nearly all (90%) overdose episodes by focusing on only 25% of the population, which greatly frees up resources for payers and patients. For those in the high- and medium-risk groups, although most will be false-positives for overdose given the overall low prevalence, additional screening and assessment may be warranted. Although certainly not perfect, these machine-learning models allow interventions to be targeted to the small number of individuals who are at greater risk, and these models are more useful than other prediction criteria that have considerably more false-positives.

### Limitations

The study has important limitations. First, patients may obtain opioids from nonmedical settings, which are not captured in claims data. Second, this study captured overdose episodes in medical settings and missed overdose episodes that occurred outside of medical settings, which are not captured in claims data. Third, the study relied on administrative billing data that lacked laboratory results and sociobehavioral information. This limitation can be addressed in the future with more robust linked data. In addition, although the study was novel in measuring overdose risk in the subsequent 3 months after initiation of prescription opioids, it used older data with complete claims capture; translation into real-time risk scores can be complicated by the lag in claims completion after the time of visit. Fourth, our focus was on predicting opioid overdose, and not opioid misuse, which is difficult to measure solely from claims data. Fifth, prediction algorithms and findings derived from the fee-for-service Medicare population may not generalize to individuals enrolled in Medicare Advantage plans or to other populations with different demographic profiles or programmatic features. However, the models may have better prediction performance in settings in which overdose is less rare (eg, Medicaid).

## Conclusions

This study demonstrates the feasibility and potential of machine-learning prediction models with routine administrative claims data available to payers. These models have high C statistics and good prediction performance and appear to be valuable tools for more accurately and efficiently identifying individuals at high risk of opioid overdose.

## Notes

Supplement. **eAppendix.** Machine Learning Approaches Used in the Study **eTable 1.** Diagnosis Codes for the Exclusion of Patients With Malignant Cancers Based on the National Committee for Quality Assurance (NCQA)’s Opioid Measures in 2018 Healthcare Effectiveness Data and Information Set (HEDIS) **eTable 2.** Diagnosis Codes for Identifying Opioid Overdose **eTable 3.** Other Diagnosis Codes Used to Identify the Likelihood of Opioid Overdose **eTable 4.** Summary of Predictor Candidates (n=268) Measured in 3-month Windows for Predicting Subsequent Opioid Overdose **eTable 5.** Opioid Overdose and Sociodemographic Characteristics Among Medicare Beneficiaries (n=560,057), Divided Into Training, Testing, and Validation Samples **eTable 6**. Prediction Performance Measures for Predicting Opioid Overdose, Across Different Machine Learning Methods With Varying Sensitivity and Specificity **eTable 7.** Comparison of Prediction Performance Using Any of Centers for Medicaid & Medicaid Services (CMS) High-Risk Opioid Use Measures vs. Deep Neural Network (DNN) and Gradient Boosting Machine (GBM) in the Validation Sample (n=166,580) Over a 12-month Period **eFigure 1**. Sample Size Flow Chart of Study Cohort **eFigure 2.** Illustrations of Two Study Designs: 3-month Windows for Measuring Predictor Candidates and Overdose Events **eFigure 3.** Classification Matrix and Definition of Prediction Performance Metrics **eFigure 4.** Prediction Performance Matrix Across Machine Learning Approaches in Predicting Opioid Overdose Risk in the Subsequent 3 Months: Sensitivity Analyses Including the Information Measured in All the Historical 3-Months Windows **eFigure 5.** Scatter Plot Between Deep Neural Network (DNN) and Gradient Boosting Machine (GBM)’s Prediction Scores **eFigure 6.** Top 50 Important Predictors for Opioid Overdose Selected by Random Forest (RF) eReferences
