# Errors in Methods, Results, Table 1, Figure 2, and Supplement

**DOI:** 10.1001/jamanetworkopen.2019.7610

**Published:** 2019-07-05

In the Original Investigation titled “Evaluation of Machine-Learning Algorithms for Predicting Opioid Overdose Risk Among Medicare Beneficiaries With Opioid Prescriptions,”^1^ published March 22, 2019, there were errors in the Methods, Results, Table 1, and Figure 2 as well as the eAppendix and eTable 7 in the Supplement. The gradient boosting machine (GBM) model should have used the top fifth percentile to calculate medium and high risk for consistency with the deep neural network (DNN) model. In the Machine-Learning Approaches and Prediction Performance Evaluation subsection of Methods, the first sentence of the third paragraph should have read, “On the basis of the distribution of individuals’ estimated probability of an overdose event, we classified beneficiaries in the validation sample into low risk (predicted score below the optimized threshold), medium risk (score between the optimized threshold and top fifth percentile), or high risk (the top fifth percentile of scores, chosen according to clinical utility).” In the first paragraph of the Risk Stratification Using Predicted Probability subsection of Results, the first 2 sentences should have read, “Using the GBM algorithm, 144 860 (77.6%) of the sample were categorized into low risk, 32 415 (17.4%) into medium risk, and 9411 (5.0%) into high risk for overdose (Table 1). Among all 91 beneficiaries with an overdose episode in the sample, 54 (59.3%) were captured in the high-risk group,” and the sixth sentence should have read, “Across both the GBM and DNN models, those in the high-risk group had 7 to 8 times the risk of overdose compared with those in the lower-risk groups (observed overdose rate of GBM: 0.57% [high risk], 0.08% [medium risk], and 0.01% [low risk]; observed overdose rate of DNN: 0.57% [high risk], 0.07% [medium risk], and 0.01% [low risk]).”

In Table 1, the columns under GBM showing medium and high risk have been replaced to show data using the top fifth percentile for calculation, and footnote “a” should have read, “Risk subgroups were classified into low risk (score below the optimized threshold), medium risk (predicted score between the optimized threshold and the top fifth percentile score), and high risk (predicted score in the top fifth percentile). The optimized thresholds were 39 (or probability of 0.39) for GBM and 46.5 (or probability of 0.465) for DNN.” In Figure 2A, the observed overdose rate should have been shown as 0.08 for medium risk and 0.57 for high risk. The caption for Figure 2 should have read, “Risk subgroups were classified into 3 groups using the optimized threshold in the validation sample (n = 186 686): low risk (score below the optimized threshold), medium risk (predicted score between the optimized threshold, identified by the Youden index, and the top fifth percentile score), and high risk (predicted score in the top fifth percentile). The dashed line indicates the overall observed overdose rate without risk stratifications.” In the eAppendix in the Supplement, the fourth paragraph of the Introduction should have shown the high-risk and medium-risk scores as using the top fifth percentile, and the final sentence of the eAppendix should have listed the probability threshold as >0.465. Finally, eTable 7 had shown data calculated using the top 10th percentile for the GBM model but using the top fifth percentile for the DNN model; data are now shown using both the top fifth and top 10th percentiles for the GBM and DNN models. This article has been corrected.^1^
