# Supplementary material for: Evaluation of Machine-Learning Algorithms for Predicting Opioid Overdose Risk Among Medicare Beneficiaries With Opioid Prescriptions
Source: JAMA Netw Open. 2019 Mar 22;2(3):e190968. doi: 10.1001/jamanetworkopen.2019.0968 (PMC6583312; doi:10.1001/jamanetworkopen.2019.0968)
Supplement: Supplement. — eAppendix. Machine Learning Approaches Used in the Study eTable 1. Diagnosis Codes for the Exclusion of Patients With Malignant Cancers Based on the National Committee for Quality Assurance (NCQA)’s Opioid Measures in 2018 Healthcare Effectiveness Data and Information Set (HEDIS) eTable 2. Diagnosis Codes for Identifying Opioid Overdose eTable 3. Other Diagnosis Codes Used to Identify the Likelihood of Opioid Overdose eTable 4. Summary of Predictor Candidates (n=268) Measured in 3-month Windows for Predicting Subsequent Opioid Overdose eTable 5. Opioid Overdose and Sociodemographic Characteristics Among Medicare Beneficiaries (n=560,057), Divided Into Training, Testing, and Validation Samples eTable 6. Prediction Performance Measures for Predicting Opioid Overdose, Across Different Machine Learning Methods With Varying Sensitivity and Specificity eTable 7. Comparison of Prediction Performance Using Any of Centers for Medicaid & Medicaid Services (CMS) High-Risk Opioid Use Measures vs. Deep Neural Network (DNN) and Gradient Boosting Machine (GBM) in the Validation Sample (n=166,580) Over a 12-month Period eFigure 1. Sample Size Flow Chart of Study Cohort eFigure 2. Illustrations of Two Study Designs: 3-month Windows for Measuring Predictor Candidates and Overdose Events eFigure 3. Classification Matrix and Definition of Prediction Performance Metrics eFigure 4. Prediction Performance Matrix Across Machine Learning Approaches in Predicting Opioid Overdose Risk in the Subsequent 3 Months: Sensitivity Analyses Including the Information Measured in All the Historical 3-Months Windows eFigure 5. Scatter Plot Between Deep Neural Network (DNN) and Gradient Boosting Machine (GBM)’s Prediction Scores eFigure 6. Top 50 Important Predictors for Opioid Overdose Selected by Random Forest (RF) eReferences [file jamanetwopen-e190968-s001.pdf]

Supplementary Online Content

Lo-Ciganic W-H, Huang JL, Zhang HH, et al. Evaluation of machine-learning algorithms for predicting opioid overdose risk among Medicare beneficiaries with opioid prescriptions. *JAMA Netw Open.* 2019;2(3):e190968. doi:10.1001/jamanetworkopen.2019.0968

eAppendix. Machine Learning Approaches Used in the Study

eTable 1. Diagnosis Codes for the Exclusion of Patients With Malignant Cancers Based on the National Committee for Quality Assurance (NCQA)'s Opioid Measures in 2018 Healthcare Effectiveness Data and Information Set (HEDIS)

eTable 2. Diagnosis Codes for Identifying Opioid Overdose

eTable 3. Other Diagnosis Codes Used to Identify the Likelihood of Opioid Overdose

eTable 4. Summary of Predictor Candidates (n=268) Measured in 3-month Windows for Predicting Subsequent Opioid Overdose

eTable 5. Opioid Overdose and Sociodemographic Characteristics Among Medicare Beneficiaries (n=560,057), Divided Into Training, Testing, and Validation Samples

eTable 6. Prediction Performance Measures for Predicting Opioid Overdose, Across Different Machine Learning Methods With Varying Sensitivity and Specificity

eTable 7. Comparison of Prediction Performance Using Any of Centers for Medicaid & Medicaid Services (CMS) High-Risk Opioid Use Measures vs. Deep Neural Network (DNN) and Gradient Boosting Machine (GBM) in the Validation Sample (n=166,580) Over a 12-month Period

eFigure 1. Sample Size Flow Chart of Study Cohort

eFigure 2. Illustrations of Two Study Designs: 3-month Windows for Measuring Predictor Candidates and Overdose Events

eFigure 3. Classification Matrix and Definition of Prediction Performance Metrics

©2019 Lo-Ciganic W-H et al. *JAMA Network Open.*

eFigure 4. Prediction Performance Matrix Across Machine Learning Approaches in Predicting Opioid Overdose Risk in the Subsequent 3 Months: Sensitivity Analyses Including the Information Measured in All the Historical 3-Months Windows

eFigure 5. Scatter Plot Between Deep Neural Network (DNN) and Gradient Boosting Machine (GBM)'s Prediction Scores

eFigure 6. Top 50 Important Predictors for Opioid Overdose Selected by Random Forest (RF)

eReferences

This supplementary material has been provided by the authors to give readers additional information about their work.

eAppendix. Machine Learning Approaches Used in the Study

Introduction

Traditional statistical model specifications require specific structural assumptions (e.g., linearity) and pre-specified factors entered in the model, which may limit opportunities for discovering new knowledge from the data, especially when model assumptions are not satisfied by real data. Machine learning is a branch of artificial intelligence in which computer algorithms learn adaptively from the data without making strict assumptions in the statistical models. The major advantages of using machine learning over traditional statistical approaches include the ability to capture non-linear and non-monotone association and to discover complex interactions that might have gone unnoticed in traditional regression models.[1] Moreover, tree-based machine learning, such as classification trees, use surrogate splits to handle missing values, whereas traditional statistical approaches require imputation for missing values, which often rely on additional assumptions on the underlying missing mechanism.

In this study, our primary goal was prediction, and the secondary goal was risk stratification (i.e., to identify subgroups of patients at similar risk of the outcome). First, we randomly and equally divided beneficiaries into training, testing, and validation samples based on the characteristic and overdose distribution. For each study design scenario (**eFigures 2A and 2B**), we developed and tested prediction algorithms for opioid overdose using 5 machine learning approaches: multivariate logistic regression, least absolute shrinkage and selection operator (LASSO), random forests (RF), gradient boosting machine (GBM), and deep neural network (DNN). For each approach, we fit the trained algorithms based on the training sample, refined the algorithm using the testing sample, and then applied the final algorithm in the validation sample to evaluate prediction performance.

Our model reporting compiles with the Standards for Reporting Diagnostic Accuracy (STARD) and the Transparent Reporting of Multivariable Prediction Model for Individual Prognosis or Diagnosis (TRIPOD) reporting guidelines.[2,3] We calculated the C-statistic (or area under the receiver operating curve [ROC]) from the validation sample to assess discrimination (i.e., the extent to which patients predicted as high-risk exhibit higher overdose rates compared to those predicted as low risk). We examined any difference in C-statistics across different approaches using the DeLong Test.[4] For each probability cutoff point, overdose was predicted for the visits with calculated probabilities above the cutoff point, whereas non-overdose was predicted for the visits with probabilities below the cutoff points. Based on their true and predicted overdose status, the patients' 3-month visits can be assigned to one of the four groups (i.e., true positive [TP], false positive [FP], true negative [TN], false negative [FN]) shown in the classification matrix (**eFigure 3**). Given that overdose events are rare outcomes and C-statistics do not incorporate information about the prevalence of the outcome, we reported other more appropriate metrics, including sensitivity, specificity, positive predictive value (PPV), negative predictive value (NPV), positive likelihood ratio (PLR), negative likelihood ratio (NLR), number needed to evaluate (NNE) to identify one overdose, and estimated rate of alerts to assess pre-implementation evaluation of our prediction algorithms (**eFigure 3**).[5] The optimal algorithm for a screening test depends on pre-test probability of the outcome, the values of TPs and TNs, and the costs of FP and FN. Since these factors vary from setting to setting (and some of them are subjective choices), no single cutoff point is suitable for every purpose. In order to compare performance across methods, we presented and assessed these prediction metrics (e.g., NNE) at the optimized threshold of the predicted probability that balances sensitivity and specificity as identified by the Youden index,[6] as well as at multiple levels of sensitivity and specificity (e.g., 90%-100%) to allow risk-benefit evaluations of interventions triggered by a positive tests using different thresholds defining high risk.

Second, based on the score (i.e., 100×predicted probability of overdose to simplify interpretation and application) in the validation sample, we classified the beneficiaries into three risk groups: low-risk (the scores below optimized threshold identified by the Youden index), high-risk (the top fifth percentile

©2019 Lo-Ciganic W-H et al. *JAMA Network Open.*

of the scores), and medium-risk (score between optimized threshold and top fifth percentile) groups. Calibration (the extent to which the predicted overdose risk agree with the observed risks) was evaluated using calibration charts of observed overdose rates by three risk group.

Multivariate logistic regression (MLR)[7]

MLR was performed with the log odds for opioid overdose as the dependent variable. The independent variables consisted of all the 268 predictor candidates and 2-way interactions between any of the two predictors. We examined correlations among the correlation coefficients to screen for multi-collinearity. Absolute values of r >0.75 were considered significant or strong correlations. We incorporated variables into a model in a stepwise fashion with α value of 0.10 to enter and 0.15 to remove. Step-wise selections were based on asymptotic covariance estimates. The goodness of fit for the final model was determined by Hosmer-Lemeshow X^2 analysis. The MLR for training visits was used to calculate a probability of overdose for each 3-month visit in the validation sample. For MLR, validation visits were assigned to one of the two predictive categories (i.e., overdose vs. non-overdose) if the probability threshold >0.01 (identified from the ROC using the Youden Index). Furthermore, we used multiple imputations (MI) with an assumption of missing at completely random for missing predictor data.[8] The MI methods simultaneously predicted missing values of variables using complete observations of variables by modeling the joint distribution of all other predictor candidates. For variables with multiple categories (e.g., provider specialty), we used multiple imputation chained equations (MICE) to impute the missing data. MLR was conducted using the software package Salford SPM v8.2.

Regularized logistic regression: Least absolute shrinkage and selection operator (LASSO)-type regularized logistic regression[9-11]

In order to simultaneously fit the regression model and select important predictors, we used several regularized or penalized logistic regression including LASSO, ridge regression, and elastic net regression, which can automatically identify factors that most strongly predicted overdose. Regularized regression uses the elastic family of penalty function for model estimation that performs variable selection and produces a parsimonious model. Briefly, after forming a prediction model with logistic regression using all candidate variables, beta coefficients are penalized and lowered to deal with model overfitting. The magnitude of penalization is subsequently changed to create various models with different prediction errors in a cross-validation process, so the final model achieves optimal penalization based on the lowest prediction error. In regularized regression, variable selection is performed automatically by shrinking regression coefficients of some variables to zero. We used multiple imputation (MI) with an assumption of missing at completely at random for missing predictor data.[8] The MI methods simultaneously predicted missing values of variables using complete observations of variables by modeling the joint distribution of all other predictor candidates. For variables with multiple categories (e.g., provider specialty), we used multiple imputation chained equations (MICE) to impute the missing data. Similar to traditional statistical methods (e.g., logistic regression), regularized regression methods cannot handle missing values and they delete rows with missing data. It also cannot discover a nonlinearity relationship or multiple interactions. However, regularized regression is expected to be more effective in the following situations: there are (1) many more columns (predictors) than rows (observations), (2) predictors available may be extremely highly correlated with each other, or (3) the goal to find the most compact model yielding acceptable performance. We used the GPS/LASSO function in the Salford SPM v8.2 to perform regularized regression with different elasticity values between 0 and 2. We used an elasticity value of 1 (which performs the LASSO regression that introduces variables as quickly as possible and produces reasonably sparse solutions), 2 (which performs the ridge regression that introduces variables sparingly and produces the least sparse

©2019 Lo-Ciganic W-H et al. *JAMA Network Open.*

solutions favoring proportional shrinkage), and 1.1 (the hybrid ridge-LASSO regression). We also controlled the amount of coefficient adjustment using the learning rate of 0.001 (learn rate between 0 and 1 and a small value forces smaller updates at each step). All other parameters were set as default values. The best model was with an elasticity value of 1.1 chosen in a cross-validation process. For regularized regression, validation visits were assigned to one of the two predictive categories (i.e., overdose vs. non-overdose) if the probability threshold >0.01 that was identified from the ROC using the Youden Index.

Random Forests (RF)[12,13]

A classification tree identifies mutually exclusive subgroups whose members share similar important predictors of the outcome of interest through a series of binary recursive partitionings.[14] RF**,** consisting of numerous independent classification trees from random sampling, improves prediction performance over a single tree.[12,15] In order to have diverse trees in the random forest, each random tree partially randomly selects a subset of predictor variables. This allows for variables of low predictive importance to enter the ensemble and potentially reveal interaction effects with other variables, which could improve overall predictive accuracy of the RF. Implementation of the tree-growing procedure in RF may be controlled by multiple tuning parameters and criteria including the number of trees in the forest, meeting *a priori* p-value thresholds to implement a binary split, the minimum sample size of observations in a node, and the number of candidate predictors to be selected at random. Practical guidance suggests that initial values for each random forest be fine-tuned iteratively by varying the value of each parameter and empirically selecting the combination of parameters to yield the lowest prediction error.[16] The tree growing process continues until a terminal node is reached, which occurs when no allowable splits exist, or stopping criteria are met. Our proposed algorithm steps and rationale were adapted from the implementation of Chirkov et al.'s RF framework.[16] We used the *Random Forests* in the software package Salford SPM for this study. For RF, validation visits were assigned to one of the two predictive categories (i.e., overdose vs. non-overdose) if the probability threshold >0.93 that was identified from the ROC using the Youden Index.

Gradient Boosting Machine (GBM; Stochastic gradient boosting or TreeNet in Salford SPM)[17,18]

Similar to RF, GBM creates a tree ensemble except that a GBM model consists of a series of trees grown in a sequential order, whereas a RF consists of a collection of trees grown in parallel. Briefly, the first tree is fitted to the data and begins with a very small tree as the initial model. The residuals (error values) from the first tree are then fed into the second tree which attempts to further reduce the error. Then it grows a second small tree to predict the residuals from the first tree. Next, it computes residuals from this new 2nd tree model and grows the 3rd tree to predict revised residuals. This process is repeated through a series of successive trees. The final predicted value is formed by adding the weighted contribution of each tree. The algorithm typically generates thousands of small decision trees built in a sequential error-correcting process to converge to an accurate model. Usually, the individual trees are fairly small (typically 3 levels deep with 6 terminal nodes), but the full GBM additive series may consist of hundreds of these small trees. Mathematically, a GBM model can be described as:

$$\textbf{GBM predicted target (i.e., opioid overdose)} = F_0 + \beta_1 * T_1(X) + \beta_2 * T_2(X) + \ldots + \beta_{100} * T_{100}(X)$$

Where F_0 is the starting value for the tree series, X is a vector of pseudo-residual values remaining at each point in the series, $T_1(X)$, $T_2(X)$, $T_3(X)$,… are trees fitted to the pseudo-residuals, and β_1, $\beta_{2,\ldots}$ are coefficients of the tree node predicted values that are computed by the GBM algorithm.

Specifically for our study, Salford's TreeNet function supplied an initial value specific to the chosen loss function (i.e. logistic binary) for each record in the training sample. TreeNet can handle

missing values automatically. We used cross entropy (i.e., negative average log likelihood) as the tuning criterion to determine the number of trees optimal for logistic models. Second, TreeNet sampled 25% of the records in the learn sample randomly and then computed the generalized residual for the records in the sample. The first tree is fitted to the data and begins with a very small tree as the initial model. TreeNet used the sampled records to fit a classification tree with a maximum 8 terminal nodes to the generalized residuals. Third, TreeNet used the classification tree derived from the sampled records to update the TreeNet model based on the loss function and shrink the update tree by the learning rate (or shrinkage rate) at 0.1 for overfitting protection. TreeNet repeated the steps previously described 200 times (i.e., number of trees to build = 200). Finally, we tested and validated the algorithms in the testing and validation samples. For TreeNet, validation visits were assigned to one of the two predictive categories (i.e., overdose vs. non-overdose) if the probability threshold >0.39, that was identified from the ROC using the Youden Index.

Deep feed-forward neural network (DNN) or Artificial neural network (ANN)[19,20]

Deep learning refers to artificial neural network (ANN) models with multiple hidden layers (typically ≥3) that can be used to quantify the complex nonlinear relationships between inputs and outputs. Recently, deep learning has shown impressive performance in image and audio processing, natural language processing, as well as biomedical fields.[19,20] Prior to conducting deep learning, we imputed missing data using median imputation for continuous variables and mode for categorical data. We also generated a series of dummy variables for categorical variables, and normalized continuous variables within a range between 0 and 1. We used a deep feed-forward neural network (DNN) to develop algorithms in our training datasets using Python 3.6 (*keras* package). We examined different numbers of hidden layers and nodes. At the end, the DNN with 2 hidden layers with 120 and 40 nodes performed the best. In this study, we generally referred our approach as DNN although the hidden layers were 2 in the best performing model. In each hidden layer during the algorithm-training process, we chose ReLU as an activation function to better prevent vanishing gradient and yield faster convergence. We applied a sigmoid function to the output layer to generate score representing the probability of overdose. We used the binary cross-entropy loss function with balanced class weight to adjust for the rare outcome. Finally, we fine-tuned the L2 regularization weight and minimized the loss function to optimize the weight and bias of the DNN. For DNN, validation visits were assigned to one of the two predictive categories (i.e., overdose vs. non-overdose) if the probability threshold >0.465 that was identified from the ROC using the Youden Index.

©2019 Lo-Ciganic W-H et al. *JAMA Network Open.*

eTable 1. Diagnosis codes for the exclusion of patients with malignant cancers based on the National Committee for Quality Assurance (NCQA)'s Opioid Measures in 2018 Healthcare Effectiveness Data and Information Set (HEDIS)

ICD-9 codes	ICD-10 codes
140.x (x=0, 1, 3, 4, 5, 6, 8, 9), 141.x (x=0, to 6, 8 and 9), 142.x (x=0, 1, 2, 8, 9), 143.x (x=0, 1, 8, 9), 144.x (x=0, 1, 8, 9), 145.x (x=0 to 6, 8, and 9), 146.x (x=0 to 9), 147.x (x=0, 1, 2, 3, 8, 9), 148.x (x=0, 1,2, 3, 8, 9), 149.x (x=0, 1, 8, 9), 150.x (x=0, 1, 2, 3, 4, 5, 8, 9), 151.x (x=0, 1, 2, 3, 4, 5, 6. 8, 9), 152.x (x=0, 1, 2, 3, 8, 9), 153.x (x=0 to 9), 154.x (x=0, 1, 2, 3, 8), 155.x (x=0, 1, 2), 156.x (x=0, 1, 2, 8, 9), 156.x (x=1, 2, 8, 9), 157.x (x=0, 1, 2, 3, 4, 8, 9), 158.x (x=0, 8, 9), 159.x (x=0, 1, 8, 9), 160.x (x=0 to 5, 8, 9), 161.x (x=0, 1, 2, 3, 8, 9), 162.x (x=0, 2, 3, 4, 5, 8, 9), 163.x (x=0, 1, 8, 9), 164.x (x=0, 1, 2, 3, 8, 9), 165.x (x=0, 8, 9), 170.x (x=0 to 9), 171.x (x=0, 2, 3, 4, 5, 6, 7, 8, 9), 172.x (x=0 to 9), 174.x (x=0 to 6, 8, 9), 175.0, 175.9, 176.x (x=0 to 5, 8, 9), 179, 180.x (x=0, 1, 8, 9), 181, 182.x (x=0, 1, 8), 183.x (x=0, 2, 3, 4, 5, 8, 9), 184.x (x=0, 1, 2, 3, 4, 8, 9), 185, 186.0, 186.9, 187.x (x=1 to 9), 188.x (x=0 to 9), 189.x (x=0, 1, 2, 3, 4, 8, 9), 190.x (x=0 to 9), 191.x (x=0 to 9), 192.x (x=0, 1, 2, 3, 8, 9), 193, 194.x (x=0, 1, 3, 4, 5, 6. 8, 9), 195.x (x=0 to 5, 8), 196.x (x=0 to 3, 5, 6, 8, 9), 197.x (x=0 to 8), 198.x (x=0 to 7), 198.81, 198.82, 198.89, 199.x (x=0, 1, 2), 200.0x (x=0 to 8), 200.1x (x=0 to 8), 200.2x (x=0 to 8), 200.3x (x=0 to 8), 200.4x (x=0 to 8), 200.5x (x=0 to 8), 200.6x (x=0 to 8), 200.7x (x=0 to 8), 200.8x (x=0 to 8), 201.0x (x=0 to 8), 201.1x (x=0 to 8), 201.2x (x=0 to 8), 201.4x (x=0 to 8), 201.5x (x=0 to 8), 201.6x (x=0 to 8), 201.7x (x=0 to 8), 201.9x (x=0 to 8), 202.0x (x=0 to 8), 202.1x (x=0 to 8), 202.2x (x=0 to 8), 202.3x (x=0 to 8), 202.4x (x=0 to 8), 202.5x (x=0 to 8), 202.6x (x=0 to 8), 202.7x (x=0 to 8), 202.8x (x=0 to 8), 202.9x (x=0 to 8), 203.0x (x=0 to 2), 203.1x (x=0 to 2), 203.8x (x=0 to 2), 204.0x (x=0 to 2), 204.1x (x=0 to 2), 204.2x (x=0 to 2), 204.8x (x=0 to 2), 204.9x (x=0 to 2), 205.0x (x=0 to 2), 205.1x (x=0 to 2), 205.2x (x=0 to 2), 205.3x (x=0 to 2), 205.8x (x=0 to 2), 205.9x (x=0 to 2), 206.0x (x=0 to 2), 206.1x (x=0 to 2), 206.2x (x=0 to 2), 206.8x (x=0 to 2), 206.9x (x=0 to 2), 207.0x (x=0 to 2), 207.1x (x=0 to 2), 207.2x (x=0 to 2), 207.8x (x=0 to 2), 208.0x (x=0 to 2), 208.1x (x=0 to 2), 208.2x (x=0 to 2), 208.8x (x=0 to 2), 208.9x (x=0 to 2), 209.0x (x=0 to 3), 209.1x (x=0 to 7), 209.2x (x=0 to 7, 9), 209.3x (x=0 to 6), 209.7x (x=0 to 5, 9).	C00.x, C01, C02.x (x=0, 1, 2, 3, 4, 8, 9), C03.x (x=0, 1, 9), C04.0, C04.1, C04.8, C04.9, C05.0, C05.1, C05.2, C05.8, C05.9, C06.0, C06.1, C06.2, C06.80, C06.89, C06.9, C07, C08.0, C08.1, C08.9, C09.0, C09.1, C09.8, C09.9, C10.x (x=0, 1, 2, 3, 4, 8, 9), C11.x (x=0, 1, 2, 3, 8, 9), C12, C13.x (x=0, 1, 2, 3, 8, 9), C14.0, C14.2, C14.8, C15.3, C15.4, C15.5, C15.8, C15.9, C16.x (x=0, 1, 2, 3, 4, 5, 6, 8, 9), C17.x (x=0, 1, 2, 3, 8, 9), C18.x, C19, C20, C21.x (x=0, 1, 2, 8), C22.x (x=0, 1, 2, 3, 4, 7, 8, 9), C23, C24.x (x=0, 1, 8, 9), C24.1, C24.8, C24.9, C25.x (x=0, 1, 2, 3, 4, 7, 8, 9), C26.0, C26.1, C26.9, C30.0, C30.1, C31.x (x=0, 1, 2, 3, 8, 9), C32.x (x=0, 1, 2, 3, 8, 9), C33, C34.00, C34.01, C34.02, C34.10, C34.11, C34.12, C34.2, C34.30, C34.31, C34.32, C34.80, C34.81, C34.82, C34.90, C34.91, C34.92, C37, C38.x (x=0, 1, 2, 3, 4, 8), C39.0, C39.9, C40.x0 (x=0, 1, 2, 3, 8, 9), C40.x1 (x=0, 1, 2, 3, 8, 9), C40.02 (x=0, 1, 2, 3, 8, 9), C41.x (x=0, 1, 2, 3, 4, 9), C43.0, C43.10, C43.11, C43.12, C43.20, C43.21, C43.22, C43.30, C43.31, C43.39, C43.4, C43.51, C43.52, C43.59, C43.60, C43.61, C43.62, C43.70, C43.71, C43.72, C43.8, C43.9, C45.0, C45.1, C45.2, C45.7, C45.9, C46.0, C46.1, C46.2, C46.3, C46.4, C46.50, C46.51, C46.52, C46.7, C46.9, C47.0, C47.10, C47.11, C47.12, C47.20, C47.21, C47.22, C47.3, C47.4, C47.5, C47.6, C47.8, C47.9, C48.0, C48.1, C48.2, C48.8, C49.0, C49.10, C49.11, C49.12, C49.20, C49.21, C49.22, C49.x (x=3, 4, 5, 6, 8, 9), C49.Ax (x=0, 1, 2,3 ,4, 5, 9), C4A.0, C4A.1x (x=0, 1, 2), C4A.2x (x=0, 1, 2), C4A.3x (x=0, 1, 9), C4A.4, C4A.5x (x=1, 2, 9), C4A.6x (x=0, 1, 2), C4A.7x (x=0, 1, 2), C4A.8, C4A.9, C50.x11 (x=0, 1, 2, 3, 4, 5, 6, 8, 9), C50.x12 (x=0, 1, 2, 3, 4, 5, 6, 8, 9), C50.x19 (x=0, 1, 2, 3, 4, 5, 6, 8, 9), C50.x21 (x=0, 1, 2, 3, 4, 5, 6, 8, 9), C50.x22 (x=0, 1, 2, 3, 4, 5, 6, 8, 9), C50.x29 (x=0, 1, 2, 3, 4, 5, 6, 8, 9), C51.x (x=0, 1, 2, 8, 9), C52, C53.x (x=0, 1, 3, 8, 9), C54.x (x=2, 3, 8, 9), C55, C56.1, C56.2, C56.9, C57.x0 (x=0, 1, 2), C57.x1 (x=0, 1, 2), C57.02 (x=0, 1, 2), C57.3, C57.4, C57.7, C57.8, C57.9, C58, C60.x (x=0, 1, 2, 8, 9), C61, C62.0x (x=0, 1, 2), C62.1x (x=0, 1, 2), C62.9x (x=0, 1, 2), C63.0x (x=0, 1, 2), C63.1x (x=0, 1, 2), C63.2, C63.7, C63.8, C63.9, C64.1, C64.2, C64.9, C65.1, C65.2, C65.9, C66.1, C66.2, C66.9, C67.x (x=0 to 9), C68.x (x=0, 1, 8, 9), C69.x0 (x=0, 1, 2, 3, 4, 5, 6, 8, 9), C69.x1 (x=0, 1, 2, 3, 4, 5, 6, 8, 9), C69.x2 (x=0, 1, 2, 3, 4, 5, 6, 8, 9), C70.0, C70.1, C70.9, C71.x, C72.0, C72.1, C72.x0 (x=2 to 5), C72.x1 (x=2 to 4), C72.x2 (x=2 to 4), C72.59, C72.9, C73, C74.0x (x=0, 1, 2), C74.1x (x=0, 1, 2), C74.9x (x=0, 1, 2), C75.x (x=0, 1, 2, 3, 4, 5, 8, 9). C76.x (x=0 to 3), C76.4x (x=0, 1, 2), C76.5x (x=0, 1, 2, 8), C77.x (x=0, 1, 2, 3, 4, 5, 8, 9), C78.0x (x=0, 1, 2), C78.1, C78.2, C78.30, C78.39, C78.4, C78.5, C78.6, C78.7, C78.80, C78.89, C79.0x (x=0, 1, 2), C79.10, C79.11, C79.19, C79.2, C79.31, C79.32, C79.40, C79.49, C79.51, C79.52, C79.6x (x=0, 1, 2), C79.7x (0, 1, 2), C79.8x (x=1, 2, 9), C79.9, C7A.00, C7A.010, C7A.011, C7A.012, C7A.019, C7A.02x (x=0 to 6, and 9), C7A.09x (x=0 to 6 and 8), C7A.1, C7A.8, C7B.0x (x=0, 1, 2, 3, 4, 9), C7B.1, C7B.8, C81.0x, C81.1x, C81.2x, C81.3x, C81.4x, C81.7x, C81.9x, C82.0x, C82.1x, C82.2x, C82.3x, C82.4x, C82.5x, C82.6x, C82.8x, C82.9x, C83.0x, C83.1x, C83.3x, C83.5x, C83.7x, C83.8x, C83.9x, C84.0x, C84.1x, C84.4x, C84.6x, C84.7x, C84.9x, C84.Ax, C84.Zx, C85.1x, C85.2x, C85.8x, C85.9x, C86.x (x=0 to 6), C88.x (x=0, 2, 3, 4, 8, 9), C90.x0 (x=0 to 3), C90.x1 (x=0 to 3), C90.x2 (x=0 to 3), C91.x0 (x=A, Z, 0, 1, 3, 4, 5, 6, 9), C91.x1 (x=A, Z, 0, 1, 3, 4, 5, 6, 9), C92.x2 (x=A, Z, 0, 1, 3, 4, 5, 6, 9), C92.9x (x=0, 1, 2), C92.Ax (x=0, 1, 2), C92.Zx (x=0, 1, 2), C93.0x (x=0, 1, 2), C93.1x (x=0, 1, 2), C93.3x (x=0, 1, 2), C93.9x (x=0, 1, 2), C93.Zx (x=0, 1, 2), C94.0x (x=0, 1, 2), C94.2x (x=0, 1, 2), C94.3x (x=0, 1, 2), C94.4x (x=0, 1, 2), C94.6, C94.8x (x=0, 1, 2), C95.0x (x=0, 1, 2), C95.1x (x=0, 1, 2), C95.9x (x=0, 1, 2), C96.x (x=0, 2, 4, 5, 6, 9, A, Z)

©2019 Lo-Ciganic W-H et al. *JAMA Network Open.*

eTable 2. Diagnosis codes for identifying opioid overdose

ICD-9	Description	ICD-10	Description
965.00	Poisoning by opium (alkaloids) unspecified	T40.0X1A	Poisoning by opium, accidental (unintentional), initial encounter
		T40.0X2A	Poisoning by opium, intentional self-harm, initial encounter
		T40.0X3A	Poisoning by opium, assault, initial encounter
		T40.0X4A	Poisoning by opium, undetermined, initial encounter
965.01	Poisoning by heroin	T40.1X1A	Poisoning by heroin, accidental (unintentional), initial encounter
		T40.1X2A	Poisoning by heroin, intentional self-harm, initial encounter
		T40.1X3A	Poisoning by heroin, assault, initial encounter
		T40.1X4A	Poisoning by heroin, undetermined, initial encounter
965.02	Poisoning by methadone	T40.3X1A	Poisoning by methadone, accidental (unintentional), initial encounter
		T40.3X2A	Poisoning by methadone, intentional self-harm, initial encounter
		T40.3X3A	Poisoning by methadone, assault, initial encounter
		T40.3X4A	Poisoning by methadone, undetermined, initial encounter
965.09	Poisoning by other opiates and related narcotics	T40.2X1A	Poisoning by other opioids, accidental (unintentional), initial encounter
		T40.2X2A	Poisoning by other opioids, intentional self-harm, initial encounter
		T40.2X3A	Poisoning by other opioids, assault, initial encounter
		T40.2X4A	Poisoning by other opioids, undetermined, initial encounter
		T40.4X1A	Poisoning by other synthetic narcotics, accidental (unintentional), initial encounter
		T40.4X2A	Poisoning by other synthetic narcotics, intentional self-harm, initial encounter
		T40.4X3A	Poisoning by other synthetic narcotics, assault, initial encounter
		T40.4X4A	Poisoning by other synthetic narcotics, undetermined, initial encounter
		T40.601A	Poisoning by unspecified narcotics, accidental (unintentional), initial encounter
		T40.602A	Poisoning by unspecified narcotics, intentional self-harm, initial encounter
		T40.603A	Poisoning by unspecified narcotics, assault, initial encounter
		T40.604A	Poisoning by unspecified narcotics, undetermined, initial encounter
		T40.691A	Poisoning by other narcotics, accidental (unintentional), initial encounter
		T40.692A	Poisoning by other narcotics, intentional self-harm, initial encounter
		T40.693A	Poisoning by other narcotics, assault, initial encounter
		T40.694A	Poisoning by other narcotics, undetermined, initial encounter
E.850.0	Accidental poisoning by heroin	N/A	N/A
E.850.1	Accidental methadone poisoning	N/A	N/A
E.850.2	Accidental opioid poisoning- not elsewhere classified.	N/A	N/A
E935.0	Heroin causing adverse effects in therapeutic use	N/A	N/A
E935.1	Methadone causing adverse effects in therapeutic use	N/A	N/A
E935.2	Other opiates and related narcotics causing adverse effects in therapeutic use	N/A	N/A

N/A: not available (i.e., no corresponding ICD-10 codes identified)

©2019 Lo-Ciganic W-H et al. *JAMA Network Open.*

eTable 3. Other diagnosis codes used to identify the likelihood of opioid overdose[a]

ICD type	ICD code	ICD codes description
**Other drug/substance-related overdose or substance use disorders**		
ICD-9	965*	Poisoning by analgesics antipyretics and anti-rheumatics
ICD-9	966	Poisoning by anticonvulsants and anti-parkinsonism drugs
ICD-9	967	Poisoning by sedatives and hypnotics
ICD-9	968	Poisoning by other central nervous system depressants and anesthetics
ICD-9	969	Poisoning by psychotropic agents
ICD-9	970	Poisoning by central nervous system stimulants
ICD-9	971	Poisoning by drugs primarily affecting the autonomic nervous system
ICD-9	972	Poisoning by agents primarily affecting the cardiovascular system
ICD-9	973	Poisoning by agents primarily affecting the gastrointestinal system
ICD-9	975	Poisoning by agents primarily acting on the smooth and skeletal muscles and respiratory system
ICD-9	977	Poisoning by other and unspecified drugs and medicinal substances
ICD-9	980	Toxic effect of alcohol
ICD-9	989	Toxic effect of other substances chiefly nonmedicinal as to source
ICD-9	303	Alcohol dependence syndrome
ICD-9	304	Drug dependence
ICD-9	305	Nondependent abuse of drugs
ICD-10	F10	Alcohol related disorders
ICD-10	F11	Opioid related disorders
ICD-10	F12	Cannabis related disorders
ICD-10	F13	Sedative, hypnotic, or anxiolytic related disorders
ICD-10	F14	Cocaine related disorders
ICD-10	F15	Other stimulant related disorders
ICD-10	F16	Hallucinogen related disorders
ICD-10	F17	Nicotine dependence
ICD-10	F18	Inhalant related disorders
ICD-10	F19	Other psychoactive substance related disorders
ICD-10	T39	Poisoning by, adverse effect of and underdosing of nonopioid analgesics, antipyretics and antirheumatics
ICD-10	T40	Poisoning by, adverse effect of and underdosing of narcotics and psychodysleptics [hallucinogens]
ICD-10	T41	Poisoning by, adverse effect of and underdosing of anesthetics and therapeutic gases
ICD-10	T42	Poisoning by, adverse effect of and underdosing of antiepileptic, sedative- hypnotic and antiparkinsonism drugs
ICD-10	T43	Poisoning by, adverse effect of and underdosing of psychotropic drugs, not elsewhere classified
ICD-10	T48	Poisoning by, adverse effect of and underdosing of agents primarily acting on smooth and skeletal muscles and the respiratory system
ICD-10	T51	Toxic effect of alcohol
ICD-10	T65	Toxic effect of other and unspecified substances

*: excluding codes for opioid and heroin overdose.
[a:] Based on Dunn KM, Saunders KW, Rutter CM, et al. Opioid prescriptions for chronic pain and overdose: a cohort study. Ann Intern Med. 2010; 152 (2):85-92 but excluding E950-959 (suicide and self-inflicted injury codes).

©2019 Lo-Ciganic W-H et al. *JAMA Network Open.*

eTable 4. Summary of predictor candidates (n=268) measured in 3-month windows for predicting subsequent opioid overdose [a]

Patterns of prescription opioid use[b]	Patterns of non-opioid prescription use	Beneficiaries sociodemographics	Health status factors	Opioid prescriber-level variables[d]	Regional-level factors[e]
• Average opioid daily dose in MME[c]<br>• Cumulative MME<br>• Cumulative duration for any opioids, SAO, and LAO<br>• Duration of longest continuous use for any opioids, SAO, and LAO<br>• No. fills of any opioids, SAO, and LAO<br>• No. standardized 30-day prescriptions for any opioids, SAO, and LAO<br>• Cumulative duration of 30-day use of any opioids, SAO, and LAO<br>• No. fills by opioid ingredient and type (e.g., any fentanyl, SAO-type fentanyl, LAO-type fentanyl)<br>• Type of opioids by Schedule and SAO/LAO (e.g., SAO, Schedule I only)<br>• No. unique opioid prescribers<br>• No. unique pharmacies<br>• No. early refills for opioids<br>• Cumulative overlapping days of early refills	• No. BZD fills<br>• No. muscle relaxants fills<br>• Cumulative overlapping days of concurrent opioid and BZD use<br>• Cumulative overlapping days of concurrent opioid and muscle relaxants use<br>• Cumulative overlapping days of concurrent opioid, BZD and muscle relaxants use<br>• Cumulative duration of buprenorphine for opioid use disorder<br>• Cumulative duration of naltrexone<br>• No. gabapentinoid fills<br>• Cumulative duration of gabapentinoid use<br>• No. antidepressants fills<br>• Cumulative duration of antidepressant use<br>• No. average monthly non-opioid prescriptions<br>• No. naltrexone fills<br>• Received methadone opioid agonist therapy[f]<br>• Received buprenorphine for OUD[f]<br>• Cumulative duration of buprenorphine therapy for OUD[f]	• Age<br>• Sex<br>• Race<br>• State of residence<br>• County of residence<br>• Zip code of residence<br>• Type of resided county (metro vs. non-metro)<br>• Disability status,<br>• Receipt of low-income subsidy	• No. outpatients visits<br>• No. ED visits<br>• No. inpatient visits<br>• History of prescription opioid overdose<br>• History of heroin overdose<br>• Days from the last opioid overdose episode<br>• History of naloxone administration<br>• Non-opioid drug use disorders<br>• Any SUD or alcohol use disorders<br>• Alcohol use disorders<br>• History of urine drug tests<br>• History of SUD counseling<br>• OUD<br>• Adjustment disorders<br>• Personality disorders<br>• Psychoses<br>• Delusional disorders<br>• Schizophrenia<br>• Mood disorders<br>• Anxiety disorders<br>• Alcohol-induced mental disorders<br>• Drug-induced mental or sleep disorders<br>• Other mental health disorders<br>• Osteoarthritis<br>• Rheumatoid arthritis<br>• Back pain<br>• Neck pain<br>• Headache or migraine<br>• Temporomandibular disorder pain<br>• Abdominal pain or hernia<br>• Chest pain<br>• Kidney or gall bladder stones<br>• Menstrual or genital reproductive pain<br>• Fractures, concussion, injuries<br>• Fibromyalgia<br>• Internal orthopedic device implant/graft<br>• Other pain conditions<br>• Surgical procedures (e.g., ischemic heart diseases)<br>• Diseases of musculoskeletal system and connective tissues<br>• Neuropathies (excluding alcoholic, drug, and optic-related)<br>• Ischemic heart disease<br>• HIV/AIDS<br>• Elixhauser index and individual categories	• Prescriber's sex<br>• Prescriber's specialties<br>• Average monthly opioid prescribing volume<br>• Average monthly opioid prescribing dose in MME<br>• Average monthly patients receiving opioids	• AHRF total health facilities variables<br>• AHRF health professions variables<br>• AHRF resource scarcity variables<br>• AHRF health training programs variables<br>• AHRF hospital expenditure, Medicare costs, VA expenditure<br>• AHRF inpatient days/discharges variables<br>• AHRF other health services utilization variables<br>• AHRF census-based variables (e.g., medium household income, employment)<br>• AHRF health insurance status variables<br>• AHRF housing statistics<br>• Area deprivation index<br>County-health ranking variables

Abbreviations: AHRF: Area Health Resources Files**; BZD:** benzodiazepines; **LAO:** long-acting opioids**; MME:** morphine milligram equivalent; **No**: Number of; **OUD:** opioid use disorder; **SAO:** short-acting opioids**; SUD:** substance use disorders;

[a:] Details for the operational definitions for each variable and corresponding diagnosis and procedure codes and National Drug Codes can be provided per request to the corresponding author.

[b:] We used an “as-prescribed” approach that assumes patients taking all prescribed opioids on the schedule recommended by their clinicians.[21] Patients who received refills for the same drug at the same dose and schedule while still having opioid prescriptions within 3 days from a prior fill were assumed to have taken the medication from the prior fill before taking medication from the second fill. (Gellad WF et al. Am J Public Health. 2018;108(2):248-255. doi: 10.2105/AJPH.2017.304174.)
[c:] We calculated morphine milligram equivalent (MME) for each opioid prescription, defined by the quantity dispensed multiplied by the strength in milligrams, multiplied by a conversion factor.[22] For each person, the average daily MME during the 3-month window was calculated by summing MMEs across all opioids and dividing by the number of days supplied.
[d:] Prescribers were identified by their National Provider Identifiers. Primary opioid prescribers were defined as the prescribers who dominantly prescribed the most opioid prescriptions. If patients only had 2 opioid prescriptions, then the first prescriber was considered as the primary prescriber.
[e]: AHRF variables (https://data.hrsa.gov/topics/health-workforce/ahrf), area deprivation index (https://www.hipxchange.org/ADI), and county-health ranking variables (http://www.countyhealthrankings.org/explore-health-rankings/use-data) are publicly available and downloadable.
f: Methadone for opioid use disorder was identified using the procedure codes (H0020, J1230) and buprenorphine for opioid use disorder was identified from prescription sublingual buprenorphine or buprenorphine/naloxone using NDC codes

©2019 Lo-Ciganic W-H et al. *JAMA Network Open.*

eTable 5. Opioid overdose and sociodemographic characteristics among Medicare beneficiaries (n=560,057), divided into training, testing, and validation samples.

Characteristic	Training (n=186,686)	Testing (n=186,685)	Validation (n=186,686)
Any opioid overdose	1,103 (0.59)	1,054 (0.56)	1,031 (0.55)
Age ≥ 65 years	119,769 (64.2)	119,675 (64.1)	119,606 (64.1)
Female	117,750 (63.1)	118,159 (63.3)	117,985 (63.2)
Race			
White	153,477 (82.2)	153, 613 (82.3)	154,083 (82.5)
Black	20,325 (10.9)	20,280 (10.9)	19,909 (10.7)
Hispanic	4,377 (2.3)	4,496 (2.4)	4,437 (2.4)
Others	8,506 (4.5)	8,297 (4.4)	8,257 (4.4)
With disability status	64,538 (34.6)	64,675 (34.6)	64,800 (34.7)
Medicaid dual eligible	75,478 (40.4)	75,949 (40.7)	76,002 (40.7)
Low income subsidy	82,296 (44.1)	82,779 (44.3)	82,760 (44.3)
End stage renal disease	58,015 (31.1)	58,061 (31.1)	57,924 (31.0)
County of residence			
Metropolitan	138,445 (74.2)	138,466 (74.2)	138,288 (74.1)
Non-metropolitan	47,971 (25.7)	47,944 (25.7)	48,159 (25.8)
Unknown	269 (0.1)	276 (0.1)	239 (0.1)

©2019 Lo-Ciganic W-H et al. *JAMA Network Open.*

eTable 6. Prediction performance measures for predicting opioid overdose, across different machine learning methods with varying sensitivity and specificity.

Methods	Score threshold (range 0-100)[a]	Predicted overdose (%)	Sensitivity (%)	Specificity (%)	PPV (%)	NPV (%)	F1 score (%)	PLR	NNE
**MLR**									
**Sensitivity**									
**100%**	**0**	**99.48**	**100**	**0.52**	**0.05**	**100**	**0.001**	1.01	**2,041**
99%	0	85.39	98.9	14.62	0.06	100	0.0011	1.16	1,771
98%	0	81.27	97.8	18.74	0.06	99.99	0.0012	1.20	1,705
97%	0	81.18	96.7	18.83	0.06	99.99	0.0012	1.19	1,722
96%	0	79.84	95.6	20.17	0.06	99.99	0.0012	1.20	1,713
95%	0	79.27	94.51	20.74	0.06	99.99	0.0012	1.19	1,721
94%	0	79.27	94.51	20.74	0.06	99.99	0.0012	1.19	1,721
93%	0	78.19	93.41	21.81	0.06	99.99	0.0012	1.19	1,717
92%	0	78.16	92.31	21.85	0.06	99.98	0.0012	1.18	1,737
91%	0	76.57	91.21	23.44	0.06	99.98	0.0012	1.19	1,722
90%	0	76.15	90.11	23.85	0.06	99.98	0.0012	1.18	1,734
**Optimized threshold[b]**	**0.22**	**11.8**	**58.24**	**88.22**	**0.24**	**99.98**	**0.0048**	**4.94**	**416**
**Specificity**									
90%	0.24	10.98	54.95	89.04	0.24	99.98	0.0049	5.01	410
91%	0.3	9	53.85	91.02	0.29	99.98	0.0058	6.00	343
92%	0.3	8.93	52.75	91.09	0.29	99.97	0.0057	5.92	347
93%	0.38	7.04	51.65	92.98	0.36	99.97	0.0071	7.36	280
94%	0.5	5.43	50.55	94.6	0.45	99.97	0.009	9.36	220
95%	0.54	4.98	48.35	95.04	0.47	99.97	0.0094	9.75	211
96%	0.65	4.13	42.86	95.89	0.51	99.97	0.01	10.43	198
97%	0.88	2.88	38.46	97.14	0.65	99.97	0.0128	13.45	154
98%	1.21	1.97	34.07	98.05	0.84	99.97	0.0165	17.47	119
99%	2.1	1	25.27	99.01	1.23	99.96	0.0235	25.53	81
**100%**	**93.25**	**0**	**0**	**100**	**0**	**99.95**	**N/A**	**N/A**	**inf**
**Maximized PPV**	**56.6**	**0**	**1.1**	**100**	**33.33**	**99.95**	**0.0213**	**N/A**	**3**
**LASSO**									
**Sensitivity**									
**100%**	**0.05**	**99.21**	**100.00**	**0.79**	**0.05**	**100.00**	**0.0010**	**1.01**	**2,035**
99%	0.05	90.09	98.90	9.91	0.05	99.99	0.0011	1.10	1,869
98%	0.05	90.09	98.90	9.91	0.05	99.99	0.0011	1.10	1,869
97%	0.05	79.33	96.70	20.67	0.06	99.99	0.0012	1.22	1,683
96%	0.05	79.33	96.70	20.67	0.06	99.99	0.0012	1.22	1,683
95%	0.05	79.33	96.70	20.67	0.06	99.99	0.0012	1.22	1,683
94%	0.05	56.34	92.31	43.68	0.08	99.99	0.0016	1.64	1,252
93%	0.05	56.34	92.31	43.68	0.08	99.99	0.0016	1.64	1,252
92%	0.05	56.34	92.31	43.68	0.08	99.99	0.0016	1.64	1,252
91%	0.05	48.93	91.21	51.09	0.09	99.99	0.0018	1.86	1,100
90%	0.05	40.04	90.11	59.99	0.11	99.99	0.0022	2.25	912
**Optimized threshold[a]**	**0.05**	**10.98**	**74.73**	**89.05**	**0.33**	**99.99**	**0.0066**	**6.82**	**301**
**Specificity**									
90%	0.05	10.07	72.53	89.96	0.35	99.99	0.0070	7.22	285
91%	0.05	9.01	68.13	91.02	0.37	99.98	0.0073	7.59	271
92%	0.05	8.03	64.84	91.99	0.39	99.98	0.0078	8.09	254
93%	0.05	7.05	61.54	92.97	0.43	99.98	0.0084	8.75	235
94%	0.05	6.02	57.14	94.00	0.46	99.98	0.0092	9.52	216
95%	0.05	5.04	53.85	94.98	0.52	99.98	0.0103	10.73	192
96%	0.05	4.01	49.45	96.01	0.60	99.97	0.0119	12.39	166
97%	0.05	3.02	43.96	97.00	0.71	99.97	0.0139	14.65	141
98%	0.05	2.01	34.07	98.00	0.82	99.97	0.0161	17.04	121
99%	0.05	1.01	23.08	99.00	1.11	99.96	0.0213	23.08	90
**100%**	**36.50**	**0.00**	**2.20**	**100.00**	**100.00**	**99.95**	**0.0430**	**N/A**	**1**
**Maximized PPV**	**36.52**	**0.00**	**1.10**	**100.00**	**100.00**	**99.95**	**0.0217**	**N/A**	**1**

©2019 Lo-Ciganic W-H et al. *JAMA Network Open.*

eTable 6 (continued).

Methods	Score threshold (range 0-100)[a]	Predicted overdose (%)	Sensitivity (%)	Specificity (%)	PPV (%)	NPV (%)	F1 score (%)	PLR	NNE
RF									
Sensitivity									
**100%**	**26.26**	**94.95**	**100**	**5.05**	**0.05**	**100**	**0.001**	**1.05**	**1,948**
99%	60.43	86.6	98.9	13.41	0.06	100	0.0011	1.14	1,796
98%	70.76	79.7	97.8	20.31	0.06	99.99	0.0012	1.23	1,672
97%	75.03	74.96	96.7	25.05	0.06	99.99	0.0013	1.29	1,590
96%	76.83	72.63	95.6	27.39	0.06	99.99	0.0013	1.32	1,558
95%	82.63	62.45	94.51	37.57	0.07	99.99	0.0015	1.51	1,356
94%	82.63	62.45	94.51	37.57	0.07	99.99	0.0015	1.51	1,356
93%	83.39	60.76	93.41	39.25	0.07	99.99	0.0015	1.54	1,335
92%	85.24	56.24	92.31	43.78	0.08	99.99	0.0016	1.64	1,250
91%	85.24	56.22	91.21	43.8	0.08	99.99	0.0016	1.62	1,264
90%	86.41	53.05	90.11	46.97	0.08	99.99	0.0017	1.70	1,208
**Optimized threshold[a]**	**92.59**	**31.86**	**79.12**	**68.16**	**0.12**	**99.99**	**0.0024**	**2.48**	**826**
Specificity									
90%	97.26	10.04	42.86	89.97	0.21	99.97	0.0041	4.27	481
91%	97.39	9.42	39.56	90.59	0.2	99.97	0.0041	4.20	489
92%	97.69	7.88	38.46	92.14	0.24	99.97	0.0047	4.89	420
93%	97.78	7.4	36.26	92.61	0.24	99.97	0.0047	4.91	419
94%	98.03	6.15	32.97	93.86	0.26	99.97	0.0052	5.37	383
95%	98.25	5.08	27.47	94.94	0.26	99.96	0.0052	5.43	379
96%	98.45	4.11	25.27	95.9	0.3	99.96	0.0059	6.16	334
97%	98.7	2.99	20.88	97.02	0.34	99.96	0.0067	7.01	294
98%	98.96	1.94	16.48	98.06	0.41	99.96	0.0081	8.49	242
99%	99.22	1	8.79	99	0.43	99.96	0.0082	8.79	234
**100%**	**99.86**	**0**	**0**	**100**	**0**	**99.95**	**N/A**	**N/A**	**INF**
**Maximized PPV**	**99.76**	**0.02**	**1.1**	**99.98**	**3.23**	**99.95**	**0.0164**	**55.0**	**31**
GBM									
Sensitivity									
**100%**	**8.01**	**91.98**	**100**	**8.02**	**0.05**	**100**	**0.0011**	**1.09**	**1,887**
99%	9.77	83.75	98.9	16.26	0.06	100	0.0012	1.18	1,737
98%	15.59	57.43	97.8	42.59	0.08	100	0.0017	1.70	1,205
97%	15.75	56.92	96.7	43.1	0.08	100	0.0017	1.70	1,208
96%	17.89	50.32	95.6	49.7	0.09	100	0.0019	1.90	1,080
95%	18.8	47.92	94.51	52.1	0.1	99.99	0.0019	1.97	1,040
94%	18.8	47.92	94.51	52.1	0.1	99.99	0.0019	1.97	1,040
93%	21.51	42.05	93.41	57.98	0.11	99.99	0.0022	2.22	924
92%	21.54	41.98	92.31	58.05	0.11	99.99	0.0021	2.20	933
91%	27.75	32.62	91.21	67.41	0.14	99.99	0.0027	2.80	734
90%	32.42	27.72	90.11	72.31	0.16	99.99	0.0032	3.25	631
**Optimized threshold[a]**	**44.54**	**18.93**	**86.81**	**81.11**	**0.22**	**99.99**	**0.0045**	**4.60**	**447**
Specificity									
90%	67.66	8.94	74.73	91.09	0.41	99.99	0.0081	8.39	245
91%	67.66	8.94	74.73	91.09	0.41	99.99	0.0081	8.39	245
92%	70.09	8.03	70.33	92	0.43	99.98	0.0085	8.79	234
93%	73.85	6.61	68.13	93.42	0.5	99.98	0.01	10.35	199
94%	75.55	5.9	63.74	94.13	0.53	99.98	0.0104	10.86	190
95%	76.83	5.38	59.34	94.65	0.54	99.98	0.0107	11.09	186
96%	80.17	4.04	57.14	95.99	0.69	99.98	0.0136	14.25	145
97%	82.48	3.05	54.95	96.98	0.88	99.98	0.0173	18.20	114
98%	84.87	2.08	45.05	97.94	1.05	99.97	0.0206	21.87	95
99%	87.72	1.02	32.97	98.99	1.57	99.97	0.03	32.64	64
**100%**	**93.83**	**0**	**0**	**100**	**0**	**99.95**	**N/A**	**N/A**	**INF**
**Maximized PPV**	**92.25**	**0.05**	**9.89**	**99.95**	**9.37**	**99.96**	**0.0963**	**197.80**	**11**

©2019 Lo-Ciganic W-H et al. *JAMA Network Open.*

eTable 6 (continued).

Methods	Score threshold (range 0-100)[a]	Predicted overdose (%)	Sensitivity (%)	Specificity (%)	PPV (%)	NPV (%)	F1 score (%)	PLR	NNE
**DNN**									
**Sensitivity**									
**100%**	**5.74**	**89.04**	**100**	**10.97**	**0.05**	**100**	**0.0011**	**1.12**	**1827**
99%	9.32	75.38	98.9	24.63	0.06	100	0.0013	1.31	1564
98%	10.27	72.29	97.8	27.73	0.07	100	0.0013	1.35	1516
97%	22.65	47.23	96.7	52.79	0.1	100	0.002	2.05	1002
96%	34.52	33.77	95.6	66.26	0.14	100	0.0028	2.83	725
95%	35.98	32.42	94.51	67.61	0.14	100	0.0028	2.92	704
94%	35.98	32.42	94.51	67.61	0.14	100	0.0028	2.92	704
93%	40.16	28.74	93.41	71.29	0.16	100	0.0032	3.25	631
92%	45.73	24.39	92.31	75.65	0.18	100	0.0037	3.79	542
91%	46.06	24.13	91.21	75.9	0.18	99.99	0.0037	3.78	543
90%	47.62	23.05	90.11	76.98	0.19	99.99	0.0038	3.91	525
**Optimized threshold[a]**	**45.73**	**24.39**	**92.31**	**75.65**	**0.18**	**100**	**0.0037**	**3.79**	**542**
**Specificity**									
90%	70.52	10.09	70.33	89.94	0.34	99.98	0.0068	6.99	294
91%	72.74	9.07	68.13	90.96	0.37	99.98	0.0073	7.54	273
92%	75.04	8.06	68.13	91.97	0.41	99.98	0.0082	8.48	243
93%	77.15	7.12	67.03	92.91	0.46	99.98	0.0091	9.45	218
94%	79.87	6.02	62.64	94.01	0.51	99.98	0.0101	10.46	197
95%	82.61	4.96	61.54	95.07	0.6	99.98	0.012	12.48	165
96%	84.95	4	59.34	96.03	0.72	99.98	0.0143	14.95	138
97%	87.44	3.06	54.95	96.97	0.88	99.98	0.0172	18.14	114
98%	90.58	1.93	50.55	98.1	1.28	99.98	0.0249	26.61	78
99%	93.48	0.99	34.07	99.02	1.67	99.97	0.0319	34.77	60
**100%**	**99.73**	**0**	**0**	**100**	**0**	**99.95**	**N/A**	**N/A**	**INF**
**Maximized PPV**	**99.62**	**0**	**1.1**	**100**	**33.33**	**99.95**	**0.0213**	**N/A**	**3**

Abbreviations: **DNN**: deep neural network; **GBM**: gradient boosting machine; **INF**: infinity; **LASSO**: least absolute shrinkage and selection operator-type regularized regression; **MLR**: multivariate logistic regression; **N/A**: not able to calculated; **NNE**: number needed to evaluate; **NPV**: negative predictive values; **PLR**: positive likelihood ratio; **PPV**: positive predictive values; **RF**: random forest.

[a]: Scores were calculated by predicted probability multiplied 100. Score threshold refers to the score used to classify or predict individuals with overdose (i.e., ≥ the threshold) vs. non-overdose (i.e., <threshold)

[b]: Optimized threshold was calculated by the Youden Index to achieve balanced sensitivity and specificity.

©2019 Lo-Ciganic W-H et al. *JAMA Network Open.*

eTable 7. Comparison of Prediction Performance Using Any of Centers for Medicaid & Medicaid Services (CMS) High-Risk Opioid Use Measures vs. Deep Neural Network (DNN) and Gradient Boosting Machine (GBM) in the Validation sample (n=166,580) over a 12 month period

eTable 7A. Using the Top 5th Percentile of Predicted Score to Define the High-Risk Groups in DNN and GBM

	GBM (using the top 5th percentile as high risk)[a]			DNN (using the top 5th percentile as high risk)[a]			CMS opioid safety measures[c]	
**Risk subgroups**	**Low (n=116,939, 70.1%)**	**Medium (n=35,812, 21.5%)**	**High (n=13,829, 8.3%)**	**Low (n=112,548, 67.6%)**	**Medium (n=38,846, 23.3%)**	**High (n=15,186, 9.1%)**	**Low or no risk opioid use (n=157,299, 94.4%)**	**High-risk opioid use (n=9,281, 5.5%)**
Medium predicted score (min, max)	15.1 (1.4, 39.0)	56.5 (39.0, 77.7)	84.8 (77.7, 93.8)	14.0 (2.1, 46.5)	62.8 (46.5, 81.9)	88.1 (81.9, 99.7)	N/A	N/A
Number of actual overdose (% of each subgroup)	8 (0.006%)	54 (0.15%)	235 (1.70%)	7 (0.006%)	21 (0.05%)	269 (1.77%)	210 (0.13%)	87 (0.93%)
No. actual non-overdose (% of each subgroup)	116,931 (99.99%)	35,758 (99.85%)	13,594 (98.3%)	112,541 (99.99%)	38,825 (99.94%)	14,917 (98.22%)	157,089 (99.86%)	9,194 (99.06%)
NNE	N/A	663	58	N/A	2,000	56	N/A	108
Overall no. misclassified (% of overall cohort)[b]	8 (0.004%)	37,758 (21.47%)	13,594 (8.16%)	7 (0.004%)	38,825 (23.3%)	14,917 (8.95%)	210 (0.12%)	9,194 (5.51%)
% of actual overdose captured among all overdose over 12 months (n=297)	2.69%	18.18%	79.12%	2.35%	7.07%	90.57%	70.7%	29.29%

Abbreviations: **N/A**: not able to calculate; **NNE:** number needed to evaluate;

[a]: Risk subgroups were classified into 3 subgroups: low-risk (below optimized score threshold), medium-risk (predicted score between the optimized score threshold and the top 5th percentile score), and high-risk (predicted score in the top 5th percentile). The optimized score thresholds are 39 (or probability of 0.39) for GBM and 46.5 (or probability of 0.465) for DNN; respectively. In contrast to Table 3, the measures are defined based on a 12 month period rather than 3 months. The sample size was smaller than in the main analysis because it required people had at least 12 months period of follow up.

[b]: If classifying medium and high-risk groups as overdose for DNN and GBM, and low-risk group as non-overdose. If classifying those with *any* of CMS high-risk opioid use measures as overdose, and the remaining will consider as non-overdose.

©2019 Lo-Ciganic W-H et al. *JAMA Network Open.*

eTable 7B. Using the top 10th percentile of predicted score to define the high-risk groups in DNN and GBM

	GBM (using the top 10th percentile as high risk)[a]			DNN (using the top 10th percentile as high risk)[a]			CMS opioid safety measures[c]	
**Risk subgroups**	**Low (n=116,939, 70.1%)**	**Medium (n=22,812, 13.6%)**	**High (n=26,829, 16.1%)**	**Low (n=112,548, 67.6%)**	**Medium (n=24,888, 14.9%)**	**High (n=29,144, 17.5%)**	**Low or no risk opioid use (n=157,299, 94.4%)**	**High-risk opioid use (n=9,281, 5.5%)**
Medium predicted score (min, max)	15.1 (1.4, 39.0)	51.4 (39.0, 62.3)	79.2 (62.3, 93.8)	14.0 (2.1, 46.5)	56.5 (46.5-67.9)	82.5 (67.9-99.7)	N/A	N/A
Number of actual overdose (% of each subgroup)	8 (0.006%)	25 (0.1%)	264 (0.98%)	7 (0.006%)	7 (0.03%)	283 (0.97%)	210 (0.13%)	87 (0.93%)
No. actual non-overdose (% of each subgroup)	116,931 (99.99%)	22,787 (99.89%)	26,565 (99.01%)	112,541 (99.99%)	24,881 (99.97%)	28,861 (99.03%)	157,089 (99.86%)	9,194 (99.06%)
NNE	N/A	1000	102	N/A	3,555	102	N/A	108
Overall no. misclassified (% of overall cohort)[b]	8 (0.004%)	22,787 (13.67%)	26,565 (15.94%)	7 (0.004%)	24,881 (14.94%)	28,861 (17.33%)	210 (0.12%)	9,194 (5.51%)
% of actual overdose captured among all overdose over 12 months (n=297)	2.69%	8.41%	88.88%	2.36%	2.36%	95.29%	70.7%	29.29%

Abbreviations: **N/A**: not able to calculate; **NNE:** number needed to evaluate;

[a]: Risk subgroups were classified into 3 subgroups: low-risk (below optimized score threshold), medium-risk (predicted score between the optimized score threshold and the top 10th percentile score), and high-risk (predicted score in the top 10th percentile). The optimized score thresholds are 39 (or probability of 0.39) for GBM and 46.5 (or probability of 0.465) for DNN; respectively. In contrast to Table 3, the measures are defined based on a 12 month period rather than 3 months. The sample size was smaller than in the main analysis because it required people had at least 12 months period of follow up.

[b]: If classifying medium and high-risk groups as overdose for DNN and GBM, and low-risk group as non-overdose. If classifying those with *any* of CMS high-risk opioid use measures as overdose, and the remaining will consider as non-overdose.

©2019 Lo-Ciganic W-H et al. *JAMA Network Open.*

eFigure 1. Sample Size Flow Chart

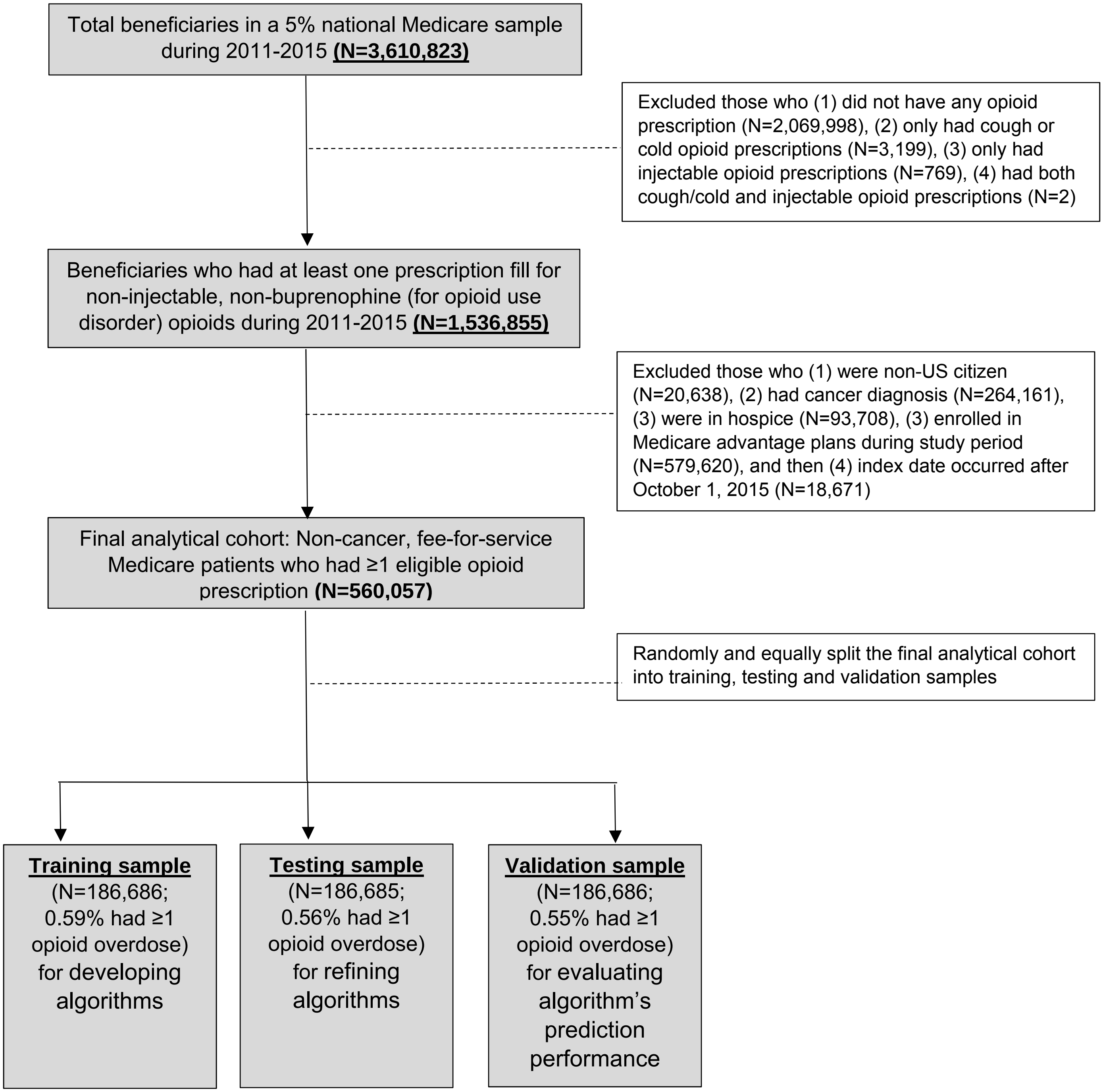

©2019 Lo-Ciganic W-H et al. *JAMA Network Open.*

eFigure 2. Illustrations two study designs: 3-month Windows for measuring predictor candidates and overdose events

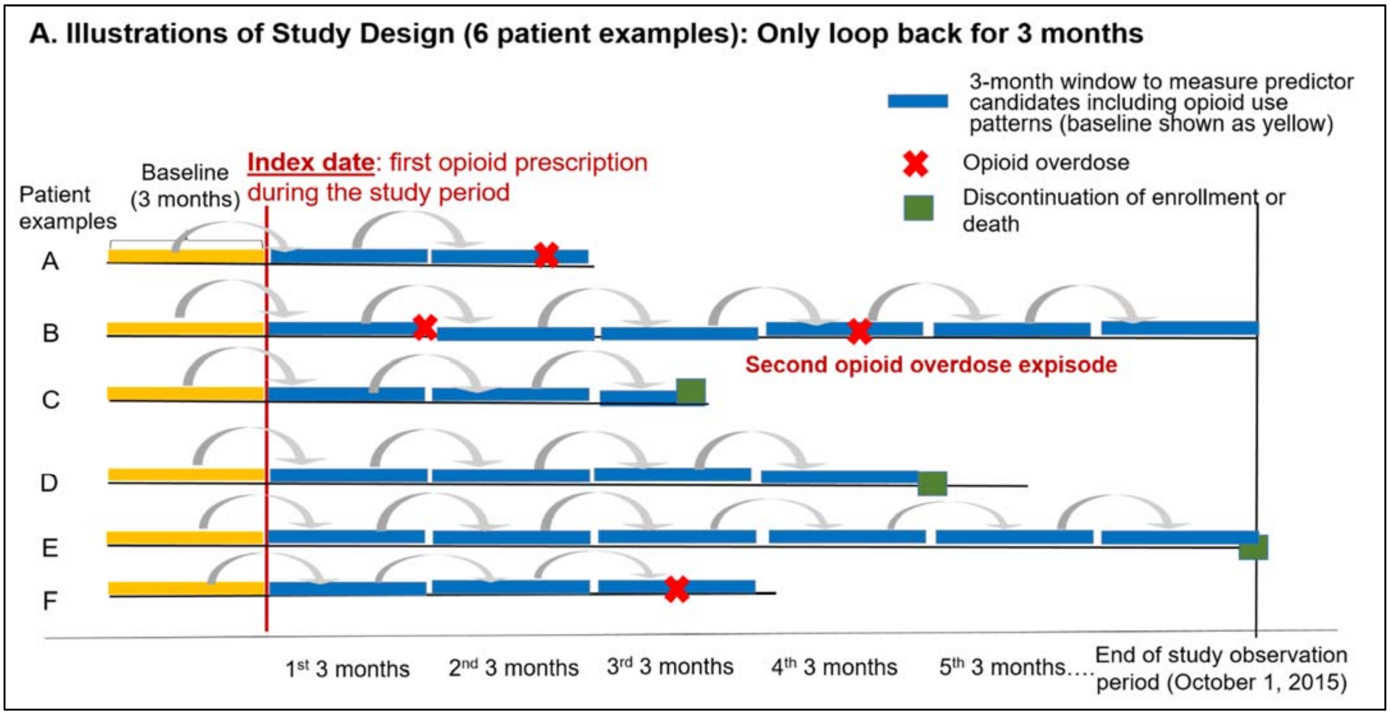

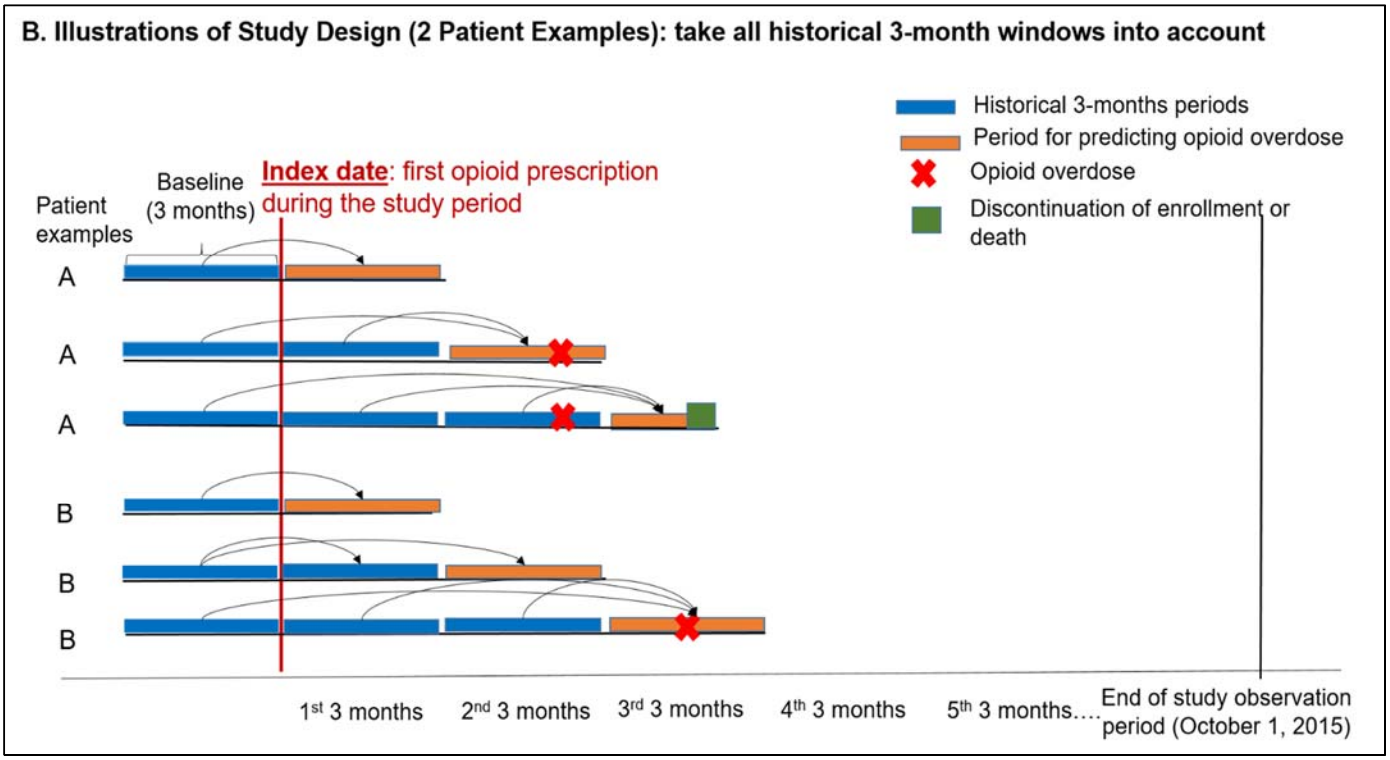

For each patient who filled at least 1 opioid prescription, we followed patients starting from 3 months before the first opioid prescriptions (i.e., the index date) and every 3 months after the index date until they were censored because of death or the end of observation. We measured predictor candidates and opioid overdose episodes for the 3-month windows. Beneficiaries might have multiple opioid overdose episodes. In eFigure 2A, we focused on 2 consecutive 3-months windows for prediction (e.g., using the factors measured in the first 3 months to predict overdose risk in the 2nd 3 months, using factors measured in the 2nd 3 months to predict overdose risk in the 3rd 3 months, …etc). In eFigure2B, we included information collected in all the historical 3-month windows to predict opioid risk for each 3-month period.

©2019 Lo-Ciganic W-H et al. *JAMA Network Open.*

eFigure 3. Classification matrix and definition of prediction performance metrics

Classification matrix	Predicted category	
Actual Category	Overdose	Non-overdose
Overdose	True positive (TP)	False negative (FN)
Non-overdose	False positive (FP)	True Negative (TN)

- $\text{Sensitivity}(S_e) \text{ or recall (Rc)} = \frac{TP}{TP+FN}$
- $\text{Specificity}(S_p) = \frac{TN}{FP+TN}$
- $\text{Positive predictive value (PPV) or precision (Pr)} = \frac{TP}{TP+FP} = \frac{\text{sensitivity}\times\text{prevalence}}{\text{sensitivity}\times\text{prevalence}+(1-\text{specificity})\times(1-\text{prevalence})}$
- $\text{Negative predictive value (NPV)} = \frac{TN}{FN+TN}$
- $\text{Positive likelihood ratio (PLR)} = \frac{\text{sensitivity}}{1-\text{specificity}}$
- $\text{Negative likelihood ratio (NLR)} = \frac{\text{specificity}}{1-\text{sensitivity}}$
- $\text{Overall misclassification ratee} = \frac{FP+FN}{TP+FN+FP+TN}$
- $\text{F1 score} = 2\frac{Pr\times Rc}{Pr+Rc}$
- $\text{Number needed to evaluate} = \frac{1}{PPV}$
- $\text{estimated rates of alerts} = TP + FP$

Prediction metrics	Definition
Sensitivity (Se) or recall (Rc)	The proportion of correctly predicted positive observations (i.e., predicted overdose) divided by all observations with actual overdose.
Specificity (Sp)	The proportion of correctly predicted negative observations (i.e., predicted non-overdose) divided by all observations with actual non-overdose.
Positive predictive value (PPV) or precision (Pr)	The proportion of actual overdose cases divided by all observations predicted as overdose. PPV is influenced by the prevalence of the outcome of interest.
Negative predictive value (NPV)	The proportion of actual non-overdose cases divided by all observations predicted as non-overdose. When the outcome is rare, NPV is typically high.
Positive likelihood ratio (PLR)	The probability that a person who has an actual overdose is predicted as overdose, divided by the probability of a person who did not have an actual overdose is predicted as overdose. The larger the PLR (>1), the better prediction performance of an algorithm.
Negative likelihood ratio (NLR)	The probability that a person who has an actual overdose predicted is predicted as non-overdose, divided by the probability that a person who did not have an actual overdose is predicted as non-overdose. The smaller the NLR (i.e., closer to 0), the better prediction performance.
Overall misclassification rate	The proportion of correctly predicted observation (i.e., actual overdose and actual non-overdose) divided by the total observations.
F1 score	The weighted average of precision (or PPV) and recall (or sensitivity). F1 takes both false positives and false negatives into account, and it usually more useful than overall misclassification rate under an uneven class distribution (e.g., non-overdose individuals comprised the majority of the cohort).[23] F1 closer to 1 is desirable.
C-statistic	The area under the receiver operating characteristics curve (ROC) curve, which is a plot of sensitivity vs. false positive (or 1-specificity) for all potential cut-off probability thresholds for an algorithm. Comparisons of C-statistics based on imbalanced data or rare outcomes can be misleading because C-statistics do not incorporate information about prevalence or pre-test probability of the outcome.[5]
Precision-recall curves	A precision-recall curve of precision (or PPV; y-axis) vs. recall (sensitivity; x-axis). The curve closer to the upper right corner (corresponding to 100% precision and 100% recall), has better performance.
Number needed to evaluate (NNE)	The NNE is the number of patients necessary to evaluate or screen to detect one outcome (i.e., overdose), similar to number needed to treat in clinical trials. A PPV of 10% is equivalent to an NNE of 10.
Estimated rate of alerts	Provides estimated number of alerts per number of patients screened or evaluated over a period of time - for example, per 100 patient per day. Too many alerts may lead to alert fatigue; too few may lead to unfamiliarity with the clinical response.

eFigure 4. Prediction performance matrix across machine learning approaches in predicting opioid overdose risk in the subsequent 3 months: sensitivity analyses including the information measured in all the historical 3-months windows

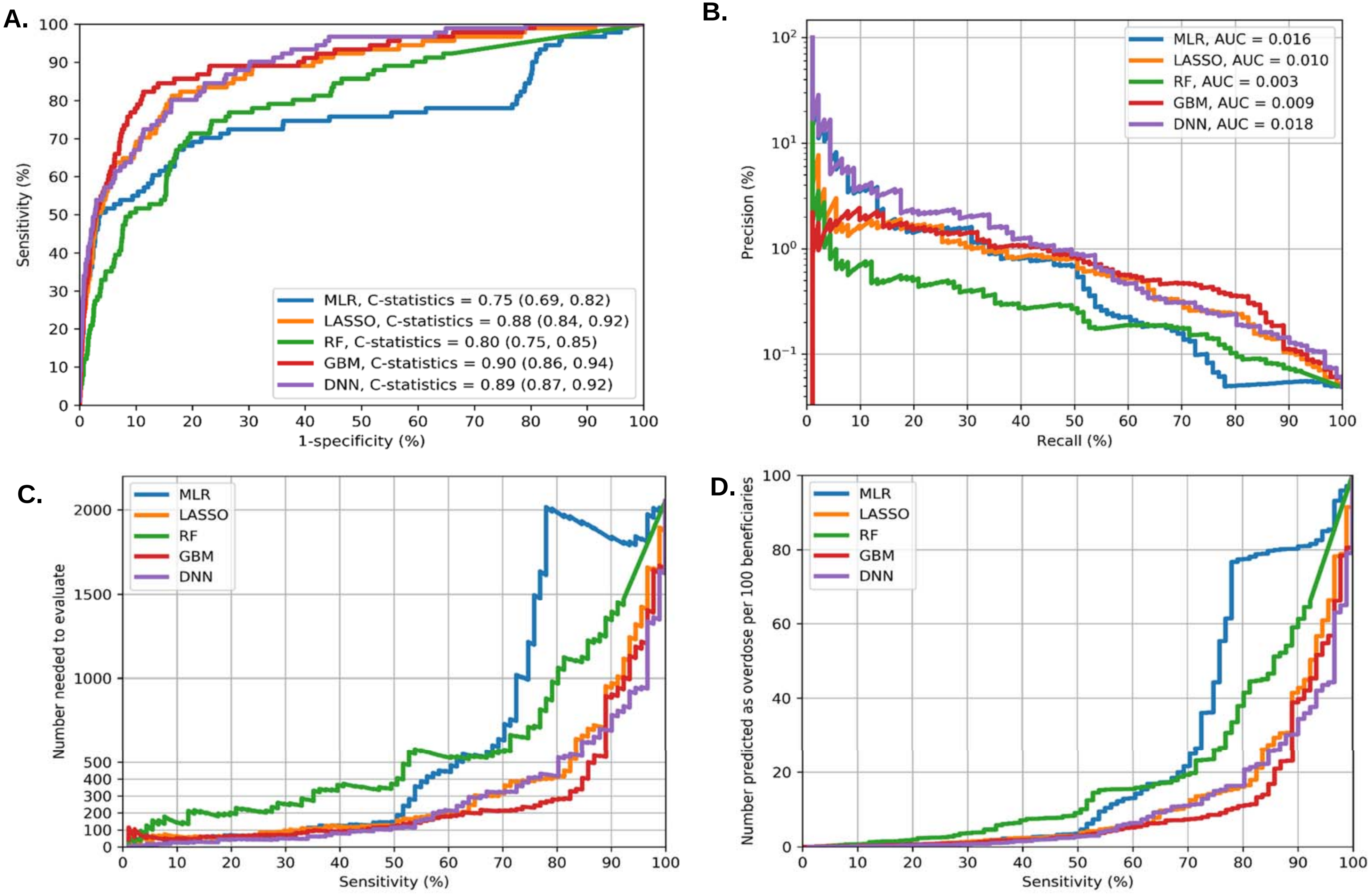

©2019 Lo-Ciganic W-H et al. *JAMA Network Open.*

Figure shows 4 prediction performance matrices in the validation sample. **eFigure 4A** shows the areas under ROC curves (or C-statistics); **eFigure 4B** shows the precision-recall curves (precision=PPV and recall=sensitivity) **-** precision recall curves that are closer to the upper right corner or above the other method have improved performance; **eFigure 4C** shows the number needed to evaluate by different cutoffs of sensitivity; and **eFigure 4D** shows alerts per 100 patients by different cutoffs of sensitivity.
Abbreviations: AUC: area under the curves; **DNN**: deep neural network; **GBM**: gradient boosting machine; **LASSO:** least absolute shrinkage and selection operator-type regularized regression**; RF**: random forest; **ROC:** Receiver Operating Characteristics.

©2019 Lo-Ciganic W-H et al. *JAMA Network Open.*

eFigure 5. Scatter plot between Deep Neural Network (DNN) and Gradient Boosting Machine (GBM)'s prediction scores

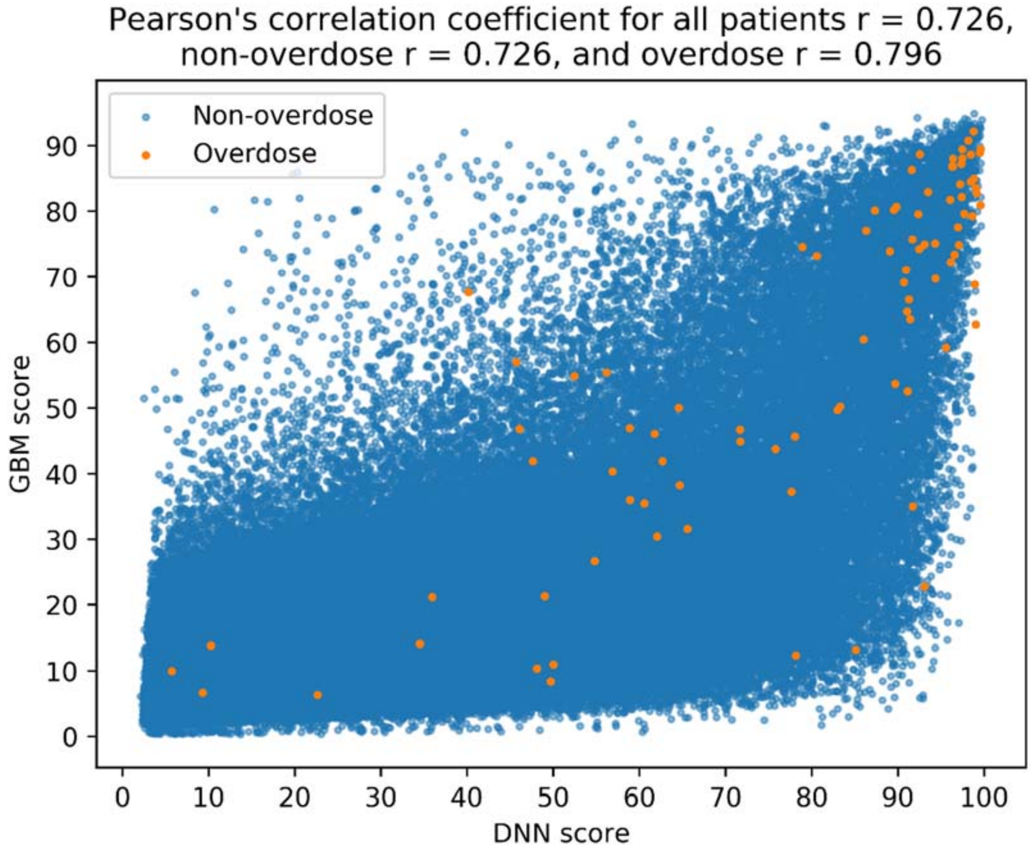

		Validation sample (n=186,686), n (% of validation sample)
Concordant prediction between GBM and DNN	DNN and GBM both predicted as non-overdose	140,313 (75.2%)
	DNN and GBM both predicted as overdose	15,973 (8.6%)
Discordant prediction between GBM and DNN	DNN predicted as non-overdose and GBM predicted as overdose	1,867 (1.0%): 3 actual overdose can be further identified from GBM
	DNN predicted as overdose and GBM predicted as non-overdose	28,533 (15.3%)

©2019 Lo-Ciganic W-H et al. *JAMA Network Open.*

eFigure 6. Top 50 important predictors for opioid overdose selected by random forest (RF)

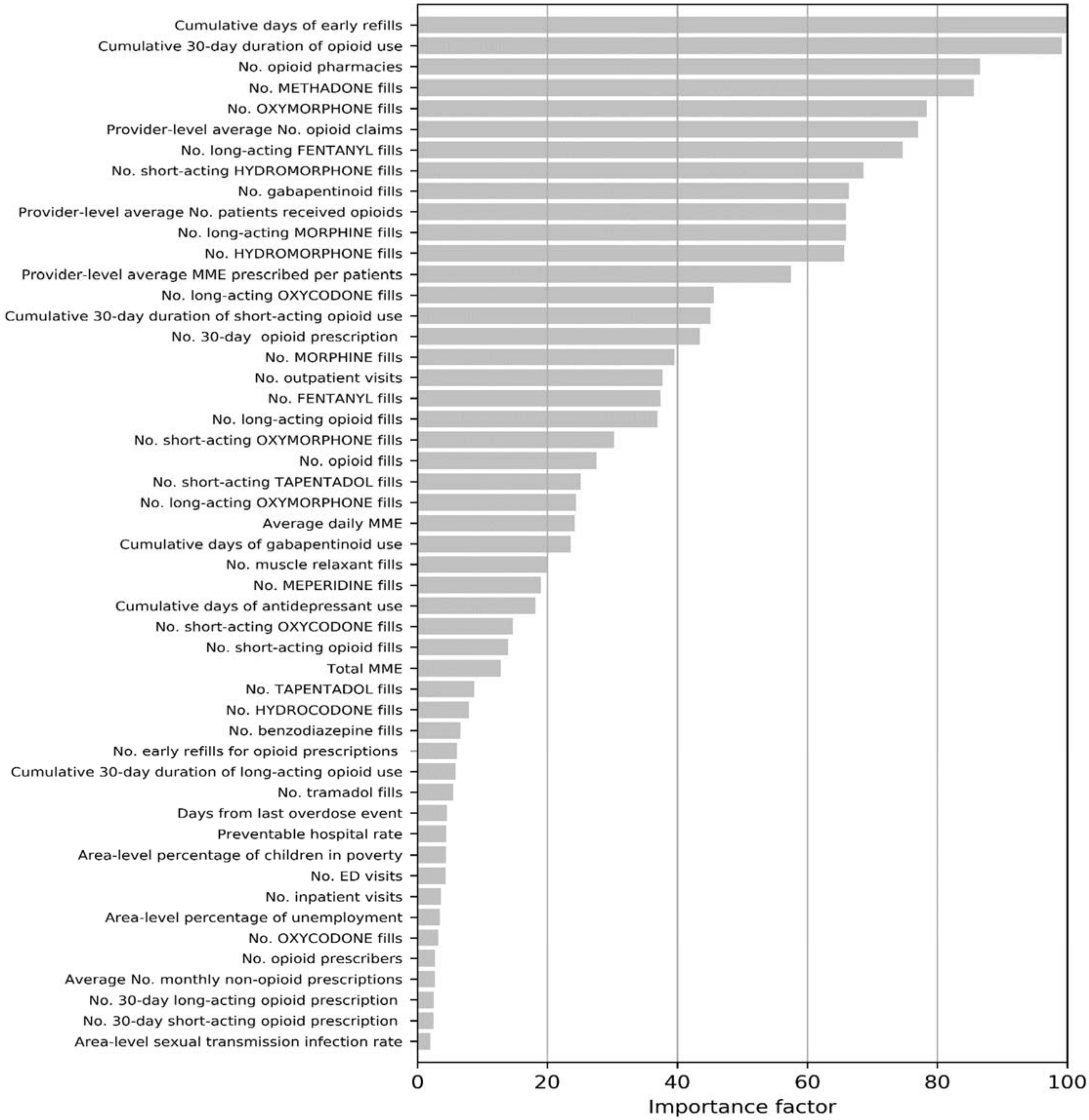

Abbreviations: **ED**: emergency department; **FFS**: fee-for-service; **MME**: morphine milligram equivalent; **No**: number of

[a] Rather than p values or coefficients, the RF reports the importance of predictor variables included in a model using both permutation-based and Gini split methods. Importance is a measure of each variable's cumulative contribution toward reducing square error, or heterogeneity within the subset, after the data set is sequentially split based on that variable. Thus, it is a reflection of a variable's impact on prediction. Absolute importance is then scaled to give relative importance, with a maximum importance of 100. For example, the top 10 important predictors identified from RF included cumulative days of early refills for opioids, cumulative 30-day duration of opioid use, No. opioid prescribing pharmacies, No. methadone fills, No. oxymorphone fills, prescriber-level average monthly opioid prescriptions, No. long-acting fentanyl fills, No. short-acting hydromorphone fills, No. gabapentinoid fills, and provider-level average monthly patients prescribed opioids.

eReferences

1. Strobl C, Malley J, Tutz G. An introduction to recursive partitioning: rationale, application, and characteristics of classification and regression trees, bagging, and random forests. *Psychol Methods.* 2009;14(4):323-348.
2. Collins GS, Reitsma JB, Altman DG, Moons KG. Transparent Reporting of a multivariable prediction model for Individual Prognosis or Diagnosis (TRIPOD): the TRIPOD statement. *Ann Intern Med.* 2015;162(1):55-63.
3. Bossuyt PM, Reitsma JB, Bruns DE, et al. STARD 2015: an updated list of essential items for reporting diagnostic accuracy studies. *BMJ.* 2015;351:h5527.
4. DeLong ER, DeLong DM, Clarke-Pearson DL. Comparing the areas under two or more correlated receiver operating characteristic curves: a nonparametric approach. *Biometrics.* 1988;44(3):837-845.
5. Romero-Brufau S, Huddleston JM, Escobar GJ, Liebow M. Why the C-statistic is not informative to evaluate early warning scores and what metrics to use. *Crit Care.* 2015;19:285.
6. Fluss R, Faraggi D, Reiser B. Estimation of the Youden Index and its associated cutoff point. *Biom J.* 2005;47(4):458-472.
7. Hosmer D, Lemeshow S. *Applied Logistic Regression.* New York1989.
8. Rubin DB, ed *Multiple Imputation for Nonresponse in Surveys.* New Yok: John Wiley and Sons; 1987.
9. Tibshirani R. Regression shrinkage and selection via the lasso. *Journal of the Royal Statistical Society Series B, Statistical methodology.* 1996;58:267-288.
10. Hastie T, Tibshirani R, Friedman J. *The Elements of Statistical Learning: : Data Mining, Inference, and Prediction.* 2nd ed. New York, NY: Springer; 2008.
11. H. FJ. Fast Sparse Regression and Classification. International Journal of Forecasting. *Int J Forecast.* 2012;28(3):722-738.
12. Breiman L. Random Forest. *Machine Learning.* 2001;45:5-32.
13. Boulesteix AL, Janitza S, Kruppa J, Konig I. Overview of random forest methodology and practical guidance with emphasis on computational biology and bioinformatics. *Wiley Interdisciplinary Reviews: Data Mining and Knowledge Discovery.* 2012;2(6):493-507.
14. Breiman L, Friedman JH, Olshen RA, Stone CJ. *Classification and regression trees.* Pacific Grove (CA): Wadsworth; 1984.
15. Ishwaran H, Kogalur UB, Blackstone EH, Lauer MS. Random survival forests. *Ann Appl Stat.* 2008;2(3):841-860.
16. Chirikov VV, Shaya FT, Onukwugha E, Mullins CD, dosReis S, Howell CD. Tree-based Claims Algorithm for Measuring Pretreatment Quality of Care in Medicare Disabled Hepatitis C Patients. *Med Care.* 2015.
17. Friedman JH. Greedy Function Approximation: A Gradient Boosting Machine. Technical report, Dept. of Statistics, Stanford University. 1999.
18. Friedman JH. A Gradient Boosting Machine. *Annals of statistics.* 2001;29(5):1189.
19. LeCun Y, Bengio Y, Hinton G. Deep learning. *Nature.* 2015;521(7553):436-444.
20. Schmidhuber J. Deep learning in neural networks: an overview. *Neural Netw.* 2015;61:85-117.
21. Bohnert AS, Valenstein M, Bair MJ, et al. Association between opioid prescribing patterns and opioid overdose-related deaths. *JAMA.* 2011;305(13):1315-1321.
22. Bohnert ASB, Valenstein M, Bair MJ, et al. Association between opioid prescribing patterns and opioid overdose-related deaths. *JAMA.* 2011;305(13):1315-1321.
23. Saito T, Rehmsmeier M. The precision-recall plot is more informative than the ROC plot when evaluating binary classifiers on imbalanced datasets. *PLoS One.* 2015;10(3):e0118432.

©2019 Lo-Ciganic W-H et al. *JAMA Network Open.*
